# Dynamic reversibility of biological aging and risk of cardiovascular disease and stroke: a longitudinal cohort study

**DOI:** 10.1007/s13167-026-00460-9

**Published:** 2026-07-02

**Authors:** Binbin Wang, Quan Li, Xizhi Tang, Xiaoqi Niu, Huakang Li

**Affiliations:** 1 Department of Cardiology, The Chenggong Hospital Affiliated to Xiamen University, Xiamen, Fujian PR China; 2 Department of Orthopedics, The Chenggong Hospital Affiliated to Xiamen University, Xiamen, Fujian PR China; 3 Department of Cardiovascular Medicine, Southwest Hospital, Army Medical University, No. 30 Gaotanyan Street, Shapingba District, Chongqing, 400038 China; 4 Key Laboratory of Geriatric Cardiovascular and Cerebrovascular Disease, Ministry of Education of China, Chongqing, China; 5 Key Laboratory of Chronobiology and Cardiometabolic Disease, Chongqing Education Commission of China, Chongqing, China

**Keywords:** Biological aging, KDM, Cardiovascular disease, Stroke, Lifestyle, Clinical risk factors, CHARLS, Predictive preventive personalised medicine (PPPM/3PM), Suboptimal health

## Abstract

**Background:**

Aging status, indexed by biological age acceleration (BAacc), is closely associated with cardiovascular disease (CVD). However, transitioning from reactive healthcare to predictive, preventive, and personalised medicine (PPPM/3PM) requires elucidating how longitudinal, dynamic transitions in aging status impact incident CVD risk.

**Methods:**

We analyzed 4,756 participants from the China Health and Retirement Longitudinal Study. Biological age was calculated using the Klemera-Doubal method (KDM). KDM-BAacc was measured at Wave 1 (baseline) and Wave 3 (follow-up) to derive multidimensional dynamic metrics: change groups, K-means clusters, aging rates, cumulative burden, and extreme phenotypes. Wave 2 was excluded from baseline calculations due to the absence of blood biomarker collection. Incident CVD (stroke and heart disease) was ascertained over a median 9.13-year follow-up. Cox models were adjusted for sociodemographic, lifestyle, and clinical risk factors.

**Results:**

Compared to sustained acceleration, reversing aging status (“accelerated to non-accelerated”) significantly lowered risks of composite CVD (HR: 0.72, 95% CI: 0.59–0.89) and stroke (HR: 0.46, 95% CI: 0.33–0.64). Consistent protective effects were observed in “high-to-low” cluster trajectories. Conversely, the “Rapid aging” phenotype (sustained highest quartile) nearly doubled stroke risk (HR: 1.97) versus the “Anti-aging” group. Faster aging rates (≥ 1) were associated with a 32% increased stroke risk, demonstrating a linear dose-response relationship. Notably, associations with heart disease were attenuated after adjusting for comorbidities.

**Conclusions:**

Biological aging is dynamically reversible, and reversing KDM-BAacc significantly mitigates CVD and stroke risks. For routine medical application, we recommend integrating longitudinal KDM-BAacc profiling into annual primary care check-ups. Crucially, these findings strongly support the paradigm shift from reactive medical services to PPPM/3PM. By identifying reversible suboptimal health prior to disease onset, this predictive tool enables targeted prevention and personalised interventions based on individual aging trajectories, decisively surpassing conventional generalized approaches.

**Supplementary Information:**

The online version contains supplementary material available at https://doi.org/10.1007/s13167-026-00460-9.

## Introduction

Population aging represents a formidable challenge to global public health. Age-related pathologies now constitute more than half of the adult disease burden worldwide, imposing economic demands for geriatric care that far outstrip those for younger demographics [1]. This demographic transition is particularly acute in China, where the proportion of individuals aged over 60 is projected to reach one-third by 2040 [2], inevitably amplifying the burden of non-communicable diseases [3]. Within this landscape, cardiovascular disease (CVD) dominates. How can healthcare systems manage the surging absolute caseload of CVD driven by an expanding elderly population, especially when age-standardized rates are paradoxically declining globally [4, 5]? The fundamental flaw in managing this escalating crisis lies in the traditional reactive medical model, which predominantly intervenes only after clinical onset. This delayed approach highlights an urgent need for a paradigm shift from reactive medical services to Predictive, Preventive, and Personalised Medicine (PPPM/3PM).

Implementing this paradigm shift requires interventions that target the transitional phase between health and disease. Clinical observations suggest that many individuals exist in suboptimal health conditions, a state characterized by reversible damage to their health, long before overt cardiovascular symptoms manifest [6]. Identifying these vulnerable individuals necessitates innovative predictive diagnostic technologies. Emerging non-invasive tools, such as tear fluid analysis [7] and continuous digital health monitoring [8], offer promising avenues for real-time health tracking. However, systemic biomarker integration remains a cornerstone of current epidemiological research. Because chronological age is an imperfect proxy for the physiological aging process, individuals sharing the same birth year often display marked divergence in disease susceptibility [9]. To capture the true extent of biological weathering, researchers have developed the concept of biological age (BA) [10]. Among various algorithms, the Klemera-Doubal method (KDM) stands out for its ability to synthesize representative biomarkers into a reliable metric [11]. The discrepancy between an individual’s biological and chronological age, termed biological age acceleration (BAacc), serves as a critical indicator of aging pace and a potent predictor of cardiovascular events [12–16].

Despite the utility of these metrics, a critical limitation of existing literature is the predominant reliance on baseline assessments, a perspective treating biological aging as a static parameter. Contemporary research challenges this view, highlighting the fluidity of the aging process. Biological aging is not a unidirectional decline but a dynamic state characterized by potential reversibility [17, 18]. At the cellular level, this reversibility is actively discussed in the context of mitochondrial biosensorics and rejuvenation, where restoring cellular bioenergetics and homeostasis can conceptually reverse aging trajectories [19, 20]. Translating these molecular concepts to population health suggests that tracking the longitudinal trajectories of BAacc could serve as a robust foundation for targeted prevention. Yet, the clinical implications of these dynamic changes, specifically whether reversing biological acceleration confers tangible protection against incident CVD and stroke, remain elusive.

### Working hypothesis in the framework of PPPM

Building upon this conceptual framework, we leveraged nationally representative longitudinal data from the China Health and Retirement Longitudinal Study (CHARLS) to address this knowledge gap. Our central hypothesis posits that biological aging is a modifiable process and that monitoring the dynamic changes of KDM-BAacc provides a powerful tool for implementing all three 3PM elements. Specifically, we hypothesize that longitudinal trajectories and distinct clustering patterns of BAacc can function as detailed predictive diagnostics to stratify CVD risk. We further postulate that reversing accelerated aging, defined as transitioning from an accelerated to a non-accelerated state, acts as an effective form of targeted prevention that significantly mitigates the risk of stroke and heart disease. Ultimately, by utilizing multidimensional metrics such as aging rate and cumulative aging burden, physicians can facilitate individualised protection against health-to-disease transition in primary care [21]. This approach allows for the personalisation of medical services based on a patient’s unique aging trajectory rather than relying on generalized clinical guidelines.

## Materials and methods

### Study design and population

This longitudinal cohort study utilized data from the CHARLS, a nationally representative survey of residents aged 45 and older across 28 provinces in China. The study protocol was approved by the Peking University Institutional Review Board (IRB00001052-11015), and written informed consent was obtained from all participants.

The CHARLS survey was conducted across five main waves: Wave 1 (2011–2012, baseline comprehensive assessment with blood sampling), Wave 2 (2013, questionnaire-based follow-up without blood sampling), Wave 3 (2015, comprehensive assessment with blood sampling), Wave 4 (2018, questionnaire-based follow-up), and Wave 5 (2020, end of current follow-up). Because the calculation of KDM-BA requires clinical biomarkers, we utilized data from Wave 1 and Wave 3. Consequently, we defined the period from Wave 1 to Wave 3 as the exposure assessment window to capture dynamic changes in biological aging. Participants were then followed up through Waves 4 and 5 to ascertain the incidence of CVD.

**Fig. 1:**
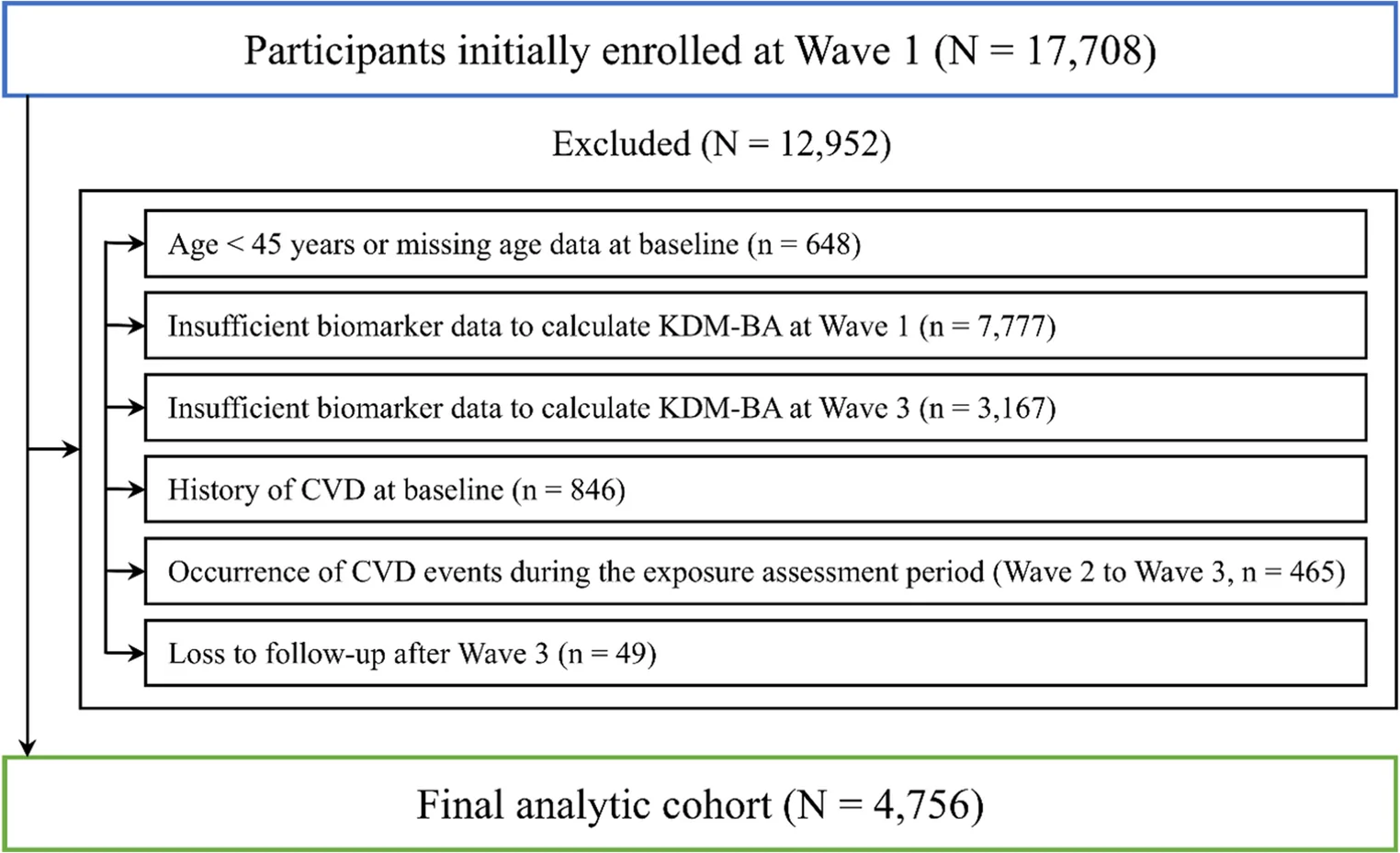
Flowchart of participant selection. Abbreviations: KDM-BA, Klemera-Doubal method biological age; CVD, cardiovascular disease

Of the 17,708 participants initially enrolled at Wave 1, we applied the following exclusion criteria sequentially: (1) age < 45 years or missing age data at baseline (*n* = 648); (2) insufficient biomarker data to calculate KDM-BA at either Wave 1 (*n* = 7,777) or Wave 3 (*n* = 3,167); (3) history of CVD at baseline (*n* = 846); (4) occurrence of CVD events during the exposure assessment period (Wave 2 to Wave 3, *n* = 465); and (5) loss to follow-up after Wave 3 (*n* = 49). The final analytic cohort comprised 4,756 participants (the flowchart of participant selection is shown in Fig. 1).

### Calculation of KDM-BA

BA was quantified using the KDM, a validated algorithm that outperforms chronological age in predicting mortality and functional decline [11, 22]. Conceptually, KDM treats chronological age as an imperfect proxy for true biological status and estimates BA by minimizing the distance between individual biomarker points and their regression lines against chronological age. We selected eight clinical biomarkers available in CHARLS that reflect cardiovascular, metabolic, renal, and immune integrity: systolic blood pressure (SBP), total cholesterol (TC), triglycerides (TG), glycated hemoglobin (HbA1c), serum creatinine, blood urea nitrogen (BUN), platelet count (PLT), and C-reactive protein (CRP). The CRP values were natural log-transformed to correct for skewness. To accommodate sexual dimorphism in physiological aging, KDM algorithms were trained separately for men and women [23]. The calculation involved a two-step process to ensure longitudinal comparability. First, we derived the regression parameters—intercept (*q*), slope (*k*), and root mean squared error (*s*)—for each biomarker by regressing them against chronological age using only the baseline (Wave 1) data. Second, these baseline-derived parameters were applied to biomarker data from both Wave 1 and Wave 3 to compute KDM-BA. This approach anchors the measurement scale to the baseline population characteristics, thereby permitting valid assessment of aging trajectories over time. Detailed calculation formulas are provided in the Supplementary Materials.

### Assessment of longitudinal biological aging dynamics

To comprehensively characterize the temporal evolution of biological aging, we constructed five distinct metrics derived from KDM-BA estimates at Wave 1 (baseline) and Wave 3 (follow-up). Categorical change in aging status: We first dichotomized KDM-BA acceleration (KDM-BAacc) at each wave, defining “Accelerated” as KDM-BAacc > 0 and “Non-accelerated” as KDM-BAacc ≤ 0. Based on the transition of these statuses over time, participants were stratified into four trajectory groups: (1) Sustained accelerated (Reference): Defined as exhibiting accelerated aging at both time points, representing a state of chronic biological aging burden. (2) Non-accelerated to accelerated: Characterized by a transition from a non-accelerated to an accelerated status, indicating a deterioration in the biological aging process. (3) Accelerated to non-accelerated: Defined as shifting from an accelerated to a non-accelerated state, suggesting biological recovery or deceleration of aging. (4) Sustained non-accelerated: Maintained a non-accelerated status throughout the follow-up period, reflecting a consistently youthful biological profile. Data-driven trajectories (k-means clustering): To identify latent patterns of aging dynamics without a priori cutoffs, we employed K-means clustering on standardized KDM-BAacc values from both waves. A four-cluster solution was specified to capture distinct longitudinal profiles, utilizing 25 random initializations to ensure stability. Clusters were semantically labeled based on centroid coordinates: “Stable low”, “Stable high”, “Low to high”, and “High to low”.

Change in age acceleration (Delta KDM-BAacc): To measure the absolute shift in aging status relative to the population mean, we calculated the difference in acceleration residuals:

$$\:{\Delta\:}BAacc=KD{M\_BA}_{acc,Wave3}-KD{M\_BA}_{acc,Wave1}$$

Positive values indicate an exacerbation of the accelerated aging phenotype, whereas negative values suggest a deceleration.

Annualized biological aging rate: We quantified the velocity of physiological decline, adjusting for variations in follow-up intervals. This metric reflects the number of biological years accrued per chronological year:

$$\:Aging\:rate=\frac{KD{M\_BA}_{Wave3}-KD{M\_BA}_{Wave1}}{Ag{e}_{Wave3}-Ag{e}_{Wave1}}$$

A rate > 1 signifies aging occurring faster than the passage of chronological time. This variable was analyzed both as a continuous measure and dichotomized at 1.0.

Cumulative aging burden: To capture the total intensity of exposure to accelerated aging across the study period, we calculated the Cumulative Age Acceleration:

$$\:Cum\:KDM\_BAacc=\frac{KD{M\_BA}_{acc,Wave1}+KD{M\_BA}_{acc,Wave3}}{2}$$

This metric was analyzed as a continuous variable and categorized into tertiles to differentiate the severity of aging burden among participants with similar trajectories. Extreme aging phenotypes: Finally, to isolate the impact of extreme biological aging patterns, we defined two specific subgroups: “Anti-aging”, comprising individuals whose KDM-BAacc fell within the lowest quartile (Q1) at both Wave 1 and Wave 3; and “Rapid aging”, comprising those whose KDM-BAacc remained in the highest quartile (Q4) at both time points.

### Outcome ascertainment

The primary endpoint of this study was incident CVD, defined as a composite outcome comprising heart disease and stroke. Consistent with established protocols in prior CHARLS investigations [24], disease status was ascertained through self-reported physician diagnoses and medication usage. Specifically, a participant was classified as having an incident heart disease event if they provided an affirmative response to either of the following inquiries: “Have you been diagnosed with a heart attack, angina, coronary heart disease, heart failure, or other heart issues?” or “Are you currently receiving any treatments (including traditional Chinese medicine, Western medicine, or other therapies) for heart disease?” A parallel methodology was employed to ascertain stroke events. Follow-up duration was calculated from the date of the baseline interview until the first occurrence of one of the following censoring events: diagnosis of the outcome of interest, death from any cause, or the administrative end of the study (August 2020), whichever occurred first. Data for participants who remained event-free were right-censored at the final follow-up.

### Covariates

To mitigate potential confounding bias, we adjusted for a comprehensive array of sociodemographic characteristics and established cardiovascular risk factors. Sociodemographic and lifestyle factors: These included age (continuous), sex (male vs. female), marital status (married/cohabitating vs. other), area of residence (urban vs. rural), educational attainment (dichotomized as below high school vs. high school and above), smoking status (yes vs. no), and alcohol consumption (yes vs. no). Body mass index (BMI) was categorized using a cutoff of 24 kg/m² (< 24 vs. ≥ 24). Clinical comorbidities: We controlled for baseline health conditions based on rigorous composite definitions combining self-reports, medication use, and biomarkers: Hypertension was identified by a self-reported diagnosis, use of antihypertensive medication, systolic blood pressure ≥ 140 mmHg, or diastolic blood pressure ≥ 90 mmHg. Diabetes mellitus was defined by self-report, use of hypoglycemic agents (including insulin), fasting plasma glucose ≥ 125 mg/dL, or HbA1c ≥ 6.5%. Dyslipidemia was ascertained through self-report, current lipid-lowering therapy, or abnormal serum lipid levels (TC ≥ 240 mg/dL, TG ≥ 150 mg/dL, low-density lipoprotein cholesterol [LDL-C] ≥ 160 mg/dL, or high-density lipoprotein cholesterol [HDL-C] < 40 mg/dL) (SI conversion factors: TC and HDL-C (mg/dL) × 0.02586 = mmol/L; TG (mg/dL) × 0.01129 = mmol/L; BUN (mg/dL) × 0.357 = mmol/L; Creatinine (mg/dL) × 88.4 = µmol/L; CRP (mg/L) × 9.524 = nmol/L). Chronic kidney disease (CKD) was determined by self-reported history, therapeutic management, or an estimated glomerular filtration rate (eGFR) < 60 ml/min/1.73 m².

## Statistical analysis

Baseline characteristics were stratified by the KDM-BAacc change groups and Cluster trajectories. Continuous variables were inspected for normality using the Kolmogorov-Smirnov test and presented as mean ± standard deviation (SD) or median (interquartile range [IQR]) as appropriate. Categorical variables were expressed as frequencies (percentages). Inter-group differences were evaluated using one-way ANOVA or the Kruskal–Wallis test for continuous data, and the Chi-square test or Fisher’s exact test for categorical data. Cox proportional hazards models were employed to estimate hazard ratios (HRs) and 95% confidence intervals (CIs) for the associations between dynamic biological aging metrics and incident CVD, stroke, and heart disease. The exposure variables included: (1) categorical trajectories (KDM-BAacc change and Cluster); (2) dichotomized change indices (Delta KDM-BAacc [< 0 vs. ≥ 0] and Aging Rate [< 1 vs. ≥ 1]); (3) Cumulative KDM-BAacc (analyzed as both a continuous variable and tertiles); and (4) Extreme phenotypes (Anti-aging vs. Rapid aging). The proportional hazards assumption was verified using Schoenfeld residuals. To control for potential confounders, we fitted three sequential models: Model 1 adjusted for sociodemographic factors (age, sex, marital status, residence, education); Model 2 further adjusted for lifestyle factors (smoking, drinking) and BMI; and Model 3 additionally included clinical comorbidities (hypertension, diabetes, dyslipidemia, CKD). Furthermore, to characterize the shape of the associations, restricted cubic splines (RCS) with four knots were utilized to visualize potential non-linear dose-response relationships between continuous aging indices (Delta KDM-BAacc, Aging Rate, and Cumulative KDM-BAacc) and outcomes.

All analyses were performed using R software, version 4.4.2 (R Foundation for Statistical Computing, Vienna, Austria). A two-sided P-value < 0.05 was considered statistically significant.

## Results

### Baseline characteristics by biological aging trajectories

In Table 1 we can see the baseline characteristics of the 4,756 participants, stratified by their longitudinal biological aging trajectories: sustained accelerated (*n* = 1,384), accelerated to non-accelerated (*n* = 813), non-accelerated to accelerated (*n* = 699), and **sustained non-accelerated** (*n* = 1,860). The mean chronological age of the cohort was 58.55 ± 8.74 years, with no significant difference observed across the four trajectory groups (*P* = 0.461). The sustained accelerated group had a higher proportion of rural residents and individuals with lower educational attainment compared to the sustained non-accelerated group. While smoking prevalence was lowest in the sustained non-accelerated group (31.3%), drinking status did not differ significantly across groups (*P* = 0.054). Participants in the sustained accelerated group exhibited a markedly worse cardiometabolic profile at baseline. They had the highest prevalence of hypertension (71.1%), diabetes (19.1%), and dyslipidemia (53.0%), as well as elevated levels of SBP, TC, and CRP compared to other groups (all *P* < 0.001). Conversely, the sustained non-accelerated group displayed the most favorable clinical parameters, characterized by lower BMI, better renal function (higher eGFR, lower BUN), and lower lipid levels.

**Table 1:** Baseline characteristics of participants stratified by biological aging trajectories

Characteristics	Total (*N* = 4756)	Sustained acc (*N* = 1384)	Acc to non-acc (*N* = 813)	Non-acc to acc (*N* = 699)	Sustained non-acc (*N* = 1860)	*P*
Age, years, mean ± SD	58.55 ± 8.74	58.83 ± 8.82	58.37 ± 8.73	58.26 ± 8.97	58.52 ± 8.59	0.461
Sex, n (%)						< 0.001
Female	2517 (52.9)	760 (54.9)	362 (44.5)	391 (55.9)	1004 (54.0)	
Male	2239 (47.1)	624 (45.1)	451 (55.5)	308 (44.1)	856 (46.0)	
Residence, n (%)						0.003
Urban	1494 (31.4)	484 (35.0)	255 (31.4)	193 (27.6)	562 (30.2)	
Rural	3262 (68.6)	900 (65.0)	558 (68.6)	506 (72.4)	1298 (69.8)	
Education level, n (%)						0.008
< High school	4379 (92.1)	1288 (93.1)	733 (90.2)	659 (94.3)	1699 (91.3)	
≥ High school	377 (7.9)	96 (6.9)	80 (9.8)	40 (5.7)	161 (8.7)	
Marital Status, n (%)						0.576
Married/cohabiting	4087 (85.9)	1204 (87.0)	698 (85.9)	595 (85.1)	1590 (85.5)	
Other	669 (14.1)	180 (13.0)	115 (14.1)	104 (14.9)	270 (14.5)	
Smoking status, n (%)						< 0.001
No	3261 (68.8)	970 (70.3)	513 (63.3)	503 (72.3)	1275 (68.7)	
Yes	1479 (31.2)	409 (29.7)	297 (36.7)	193 (27.7)	580 (31.3)	
Drinking status, n (%)						0.054
No	3098 (65.2)	922 (66.7)	496 (61.1)	459 (65.8)	1221 (65.7)	
Yes	1654 (34.8)	461 (33.3)	316 (38.9)	239 (34.2)	638 (34.3)	
BMI, kg/m², mean ± SD	23.44 ± 3.77	24.39 ± 4.09	23.34 ± 3.55	23.28 ± 3.60	22.83 ± 3.54	< 0.001
BMI Categories, n (%)						< 0.001
< 24 kg/m²	2877 (61.1)	690 (50.2)	497 (61.7)	429 (62.1)	1261 (68.5)	
≥ 24 kg/m²	1835 (38.9)	684 (49.8)	309 (38.3)	262 (37.9)	580 (31.5)	
SBP, mmHg, mean ± SD	127.74 ± 20.42	140.27 ± 21.38	133.38 ± 20.13	121.75 ± 15.30	118.22 ± 15.05	< 0.001
Hypertension, n (%)	2141 (46.0)	975 (71.1)	457 (56.9)	254 (37.6)	455 (25.2)	< 0.001
Diabetes, n (%)	666 (14.0)	265 (19.1)	112 (13.8)	96 (13.7)	193 (10.4)	< 0.001
Dyslipidemia, n (%)	2223 (46.7)	733 (53.0)	388 (47.7)	335 (47.9)	767 (41.2)	< 0.001
CKD, n (%)	315 (6.6)	119 (8.6)	59 (7.3)	44 (6.3)	93 (5.0)	< 0.001
TC, mg/dL	193.04 ± 37.72	199.76 ± 40.12	196.85 ± 38.05	190.57 ± 36.55	187.30 ± 35.11	< 0.001
TG, mg/dL, median (IQR)	103.54 (73.5, 151.3)	107.08 (77.0, 161.1)	99.12 (70.8, 144.3)	107.08 (76.1, 160.2)	100.89 (73.2, 144.3)	< 0.001
HDL-C, mg/dL	51.41 ± 15.27	50.55 ± 15.18	52.72 ± 15.42	51.57 ± 15.68	51.42 ± 15.07	0.015
HbA1c, %	5.25 ± 0.80	5.39 ± 1.05	5.27 ± 0.65	5.21 ± 0.69	5.16 ± 0.64	< 0.001
Creatinine, mg/dL	0.76 ± 0.17	0.84 ± 0.19	0.82 ± 0.17	0.73 ± 0.16	0.70 ± 0.14	< 0.001
BUN, mg/dL	15.65 ± 4.29	17.43 ± 4.62	17.27 ± 4.56	14.63 ± 3.56	14.01 ± 3.29	< 0.001
eGFR, mL/min/1.73 m²	97.32 ± 12.96	91.49 ± 14.85	94.83 ± 12.96	100.00 ± 11.28	101.75 ± 9.69	< 0.001
CRP, mg/L, median (IQR)	0.96 (0.53, 1.96)	1.36 (0.74, 2.78)	1.28 (0.65, 2.88)	0.78 (0.47, 1.52)	0.71 (0.44, 1.35)	< 0.001
Platelets, 10^9^/L	211.27 ± 71.60	204.14 ± 71.71	201.88 ± 68.97	219.01 ± 72.36	217.77 ± 71.43	< 0.001
KDM-BA, years	58.40 ± 9.24	61.52 ± 9.17	59.94 ± 8.98	56.65 ± 9.05	56.07 ± 8.65	< 0.001
KDM-BAacc, median (IQR)	-0.26 (-2.00, 1.53)	2.19 (0.98, 3.86)	1.20 (0.53, 2.31)	-1.22 (-2.28, -0.55)	-2.11 (-3.44, -1.06)	< 0.001
Aging rate, median (IQR)	1.06 (0.64, 1.47)	1.09 (0.73, 1.40)	0.39 (0.03, 0.63)	1.82 (1.53, 2.24)	1.07 (0.80, 1.36)	< 0.001
Δ KDM-BAacc, median (IQR)	-0.10 (-1.76, 1.57)	0.03 (-1.40, 1.26)	-2.77 (-4.21, -1.78)	2.97 (1.79, 4.71)	-0.08 (-1.15, 1.09)	< 0.001
Cum KDM-BAacc, median (IQR)	-0.26 (-1.80, 1.31)	2.23 (1.29, 3.58)	-0.01 (-0.56, 0.55)	0.14 (-0.44, 0.74)	-2.17 (-3.26, -1.34)	< 0.001
Heart disease, n (%)	667 (14.0)	224 (16.2)	108 (13.3)	113 (16.2)	222 (11.9)	0.002
Stroke, n (%)	352 (7.4)	148 (10.7)	39 (4.8)	58 (8.3)	107 (5.8)	< 0.001
CVD, n (%)	931 (19.6)	339 (24.5)	137 (16.9)	150 (21.5)	305 (16.4)	< 0.001

*BMI* body mass index, *SBP* systolic blood pressure, *CKD* chronic kidney disease, *TC* total cholesterol, *TG* triglycerides, *HDL-C* high-density lipoprotein cholesterol, *HbA1c* glycated hemoglobin, *BUN* blood urea nitrogen, *eGFR* estimated glomerular filtration rate, *CRP* C-reactive protein, *KDM-BA* Klemera-Doubal method biological age; *Acc* accelerated, *KDM-BAacc* KDM biological age acceleration, *Cum KDM-BAacc* cumulative KDM biological age acceleration, *CVD* cardiovascular disease, *SD* standard deviation, *IQR* interquartile range. Data are presented as mean ± SD for normally distributed continuous variables, median (IQR) for skewed continuous variables, and n (%) for categorical variables. *P*-values were derived from one-way ANOVA or Kruskal-Wallis tests for continuous variables and Chi-square tests for categorical variables. SI conversion factors: TC and HDL-C (mg/dL) × 0.02586 = mmol/L; TG (mg/dL) × 0.01129 = mmol/L; BUN (mg/dL) × 0.357 = mmol/L; Creatinine (mg/dL) × 88.4 = µmol/L; CRP (mg/L) × 9.524 = nmol/L

As expected, biological aging indicators varied significantly by group definition. Although chronological age was similar, the baseline KDM-BA was highest in the sustained accelerated group (61.52 ± 9.17 years) and lowest in the sustained non-accelerated group (56.07 ± 8.65 years). notably, the non-accelerated to accelerated group, despite starting with a negative KDM-BAacc at baseline, exhibited the fastest rate of aging during follow-up (aging rate median: 1.82), whereas the accelerated to non-accelerated group showed a substantial deceleration (aging rate median: 0.39). During a median follow-up of 9.13 years, the incidence of composite CVD events was significantly higher in the sustained accelerated (24.5%) and non-accelerated to accelerated (21.5%) groups compared to the sustained non-accelerated (16.4%) and accelerated to non-accelerated (16.9%) groups (*p* < 0.001). a similar pattern was observed for stroke and heart disease individually, with the sustained accelerated group consistently showing the highest burden of disease.

**Fig. 2:**
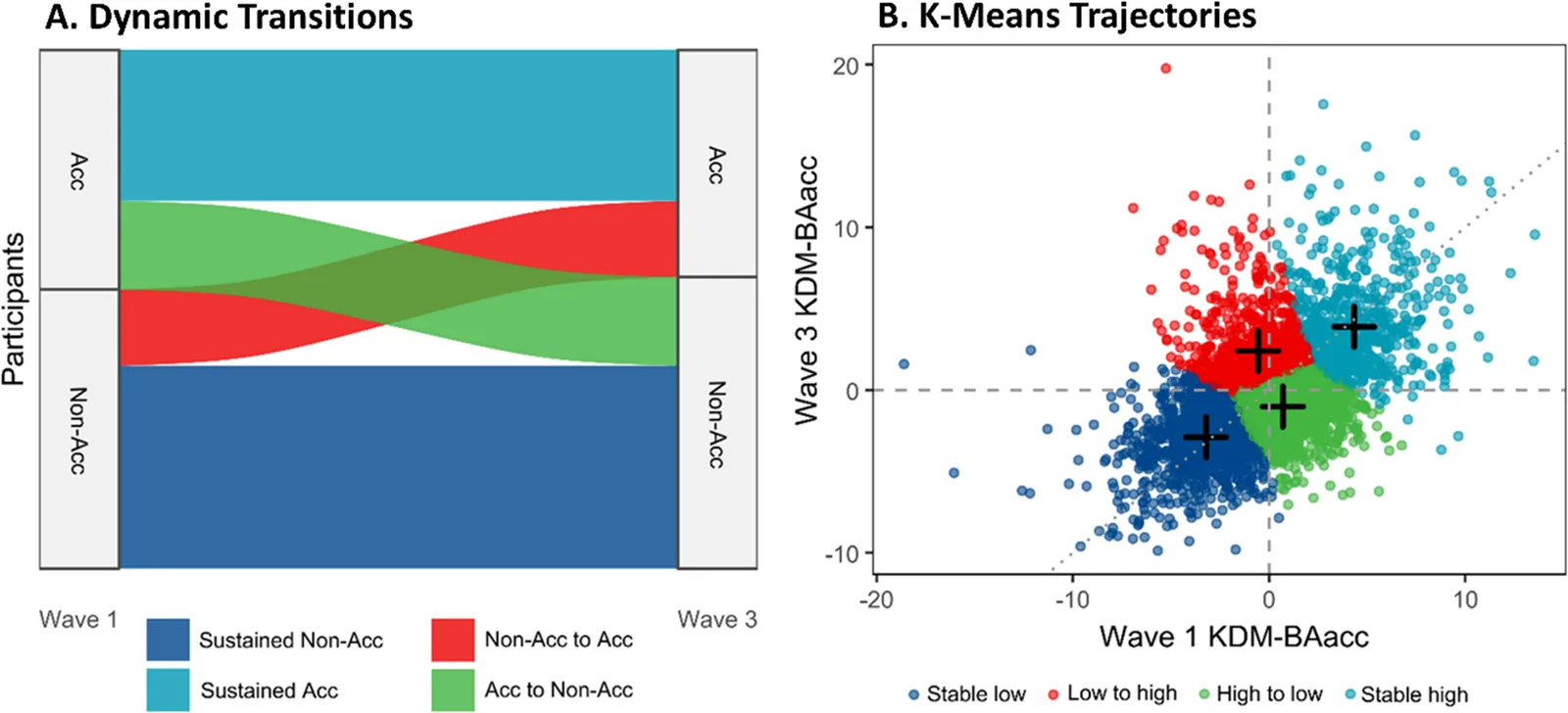
Dynamic transitions and K-means clustering trajectories of KDM-BAacc from Wave 1 to Wave 3. (**A**) Dynamic Transitions: The Sankey diagram illustrates the longitudinal transitions of biological aging status between Wave 1 and Wave 3. Participants were categorized into “Accelerated” (Acc, KDM-BAacc > 0) and “Non-accelerated” (Non-Acc, KDM-BAacc ≤ 0) groups at each wave. Four distinct transition patterns were identified: Sustained accelerated (cyan), sustained non-accelerated (blue), non-accelerated to accelerated (red), accelerated to non-accelerated (green). (**B**) K-Means Trajectories: Scatter plot showing the distribution of KDM-BAacc at Wave 1 (x-axis) and Wave 3 (y-axis). K-means clustering analysis identified four clusters representing different aging trajectories, consistent with the transition patterns: Stable high (cyan dots), stable low (blue dots), low to high (red dots), high to low (green dots). The black crosses (+) represent the centroids of each cluster. The dashed lines indicate the zero-intercepts for both waves, and the diagonal dotted line represents the line of stability (y = x). Abbreviations: KDM-BAacc, Klemera-Doubal method biological age acceleration; Acc, accelerated; Non-Acc, non-accelerated

K-means clustering (Fig. 2B) aligned well with the transition patterns, yielding four distinct groups. While Wave 1 and Wave 3 KDM-BAacc were positively correlated, the scatter plot highlighted substantial trajectory shifts among many individuals. As shown in Table S1, the four clusters identified by K-means analysis exhibited distinct baseline profiles, the stable high cluster represented the most metabolically compromised group, with the highest BMI, blood pressure, and levels of glucose, lipids, and CRP, as well as the highest incidence of CVD (27.0%). In contrast, the stable low cluster showed the healthiest baseline parameters. Notably, the low to high cluster, despite having a moderate baseline risk, exhibited the fastest rate of biological aging acceleration during the study period (median aging rate: 1.63), which corresponded to the second-highest CVD incidence (22.9%).

### Association of biological aging trajectories and clusters with incident CVD

The data in Table 2 present the HRs for incident cardiovascular outcomes associated with longitudinal biological aging trajectories, fully adjusted for sociodemographic and clinical covariates (Model 3). Using the sustained accelerated group as the reference, participants who maintained a non-accelerated aging status (sustained non-accelerated) exhibited a significantly lower risk of composite CVD (HR: 0.77, 95% CI: 0.65–0.92, *P* = 0.003). Notably, reversing biological aging acceleration conferred a substantial protective effect; the accelerated to non-accelerated group showed a 28% reduction in CVD risk (HR: 0.72, 95% CI: 0.59–0.89, *P* = 0.002). Conversely, participants who transitioned from a non-accelerated to an accelerated state had a CVD risk comparable to those with sustained accelerated (HR: 0.98, *P* = 0.875). Analyses based on K-means clustering yielded highly consistent results: compared to the stable high cluster, both the stable low (HR: 0.77) and high to low (HR: 0.73) clusters were associated with significantly reduced CVD risks, whereas the low to high cluster showed no significant risk difference (HR: 0.98, *P* = 0.860).

**Table 2:** Associations of biological aging trajectories and clusters with the risk of CVD

Groups	CVD HR (95% CI)	Stroke HR (95% CI)	Heart disease HR (95% CI)
Longitudinal trajectories			
Sustained acc	1.00 (Reference)	1.00 (Reference)	1.00 (Reference)
Sustained non-acc	0.77 (0.65 ~ 0.92) **	0.63 (0.47 ~ 0.82) ***	0.86 (0.70 ~ 1.06)
Non-acc to acc	0.98 (0.80 ~ 1.21)	0.85 (0.62 ~ 1.18)	1.16 (0.91 ~ 1.47)
Acc to non-acc	0.72 (0.59 ~ 0.89) **	0.46 (0.32 ~ 0.65) ***	0.89 (0.70 ~ 1.12)
K-means clusters			
Stable high	1.00 (Reference)	1.00 (Reference)	1.00 (Reference)
Stable low	0.77 (0.62 ~ 0.96) *	0.58 (0.41 ~ 0.82) **	0.89 (0.68 ~ 1.15)
Low to high	0.98 (0.80 ~ 1.21)	0.83 (0.60 ~ 1.14)	1.15 (0.90 ~ 1.47)
High to low	0.73 (0.60 ~ 0.89) **	0.53 (0.39 ~ 0.73) ***	0.88 (0.69 ~ 1.11)

*HR* hazard ratio, *CI* confidence interval, *CVD* cardiovascular disease, *BMI* body mass index, *CKD* chronic kidney disease, *Acc* accelerated, *Non-acc* non-accelerated. Models were adjusted for age, sex, residence, education level, marital status, smoking status, drinking status, BMI, and baseline chronic conditions (hypertension, diabetes, dyslipidemia, CKD). *p < 0.05; **p < 0.01; ***p < 0.001

When examining specific CVD subtypes, the associations were driven primarily by stroke. As shown in Table2 the risk of stroke was markedly lower in the sustained non-accelerated (HR: 0.63, *P* < 0.001) and accelerated to non-accelerated (HR: 0.46, *P* < 0.001) groups compared to the reference. Similarly, the stable low and high to low clusters showed robust protective effects against stroke (HR: 0.58 and 0.53, respectively). In contrast, for heart disease, although protective trends were observed in unadjusted and partially adjusted models (Table S2), no statistically significant associations remained in the fully adjusted Model 3 for either the trajectory-based or cluster-based groups (*P* > 0.05), suggesting that the impact of biological aging on heart disease may be largely mediated by traditional clinical risk factors included in the model. Table S2 details the stepwise attenuation of risk estimates. Significant associations with heart disease observed in Model 1 and Model 2 were rendered non-significant after further adjustment for lifestyle factors and comorbidities (hypertension, diabetes, dyslipidemia, and CKD) in Model 3, highlighting the confounding role of these clinical variables. However, the associations with composite CVD and stroke remained robust and statistically significant across all models.

**Fig. 3:**
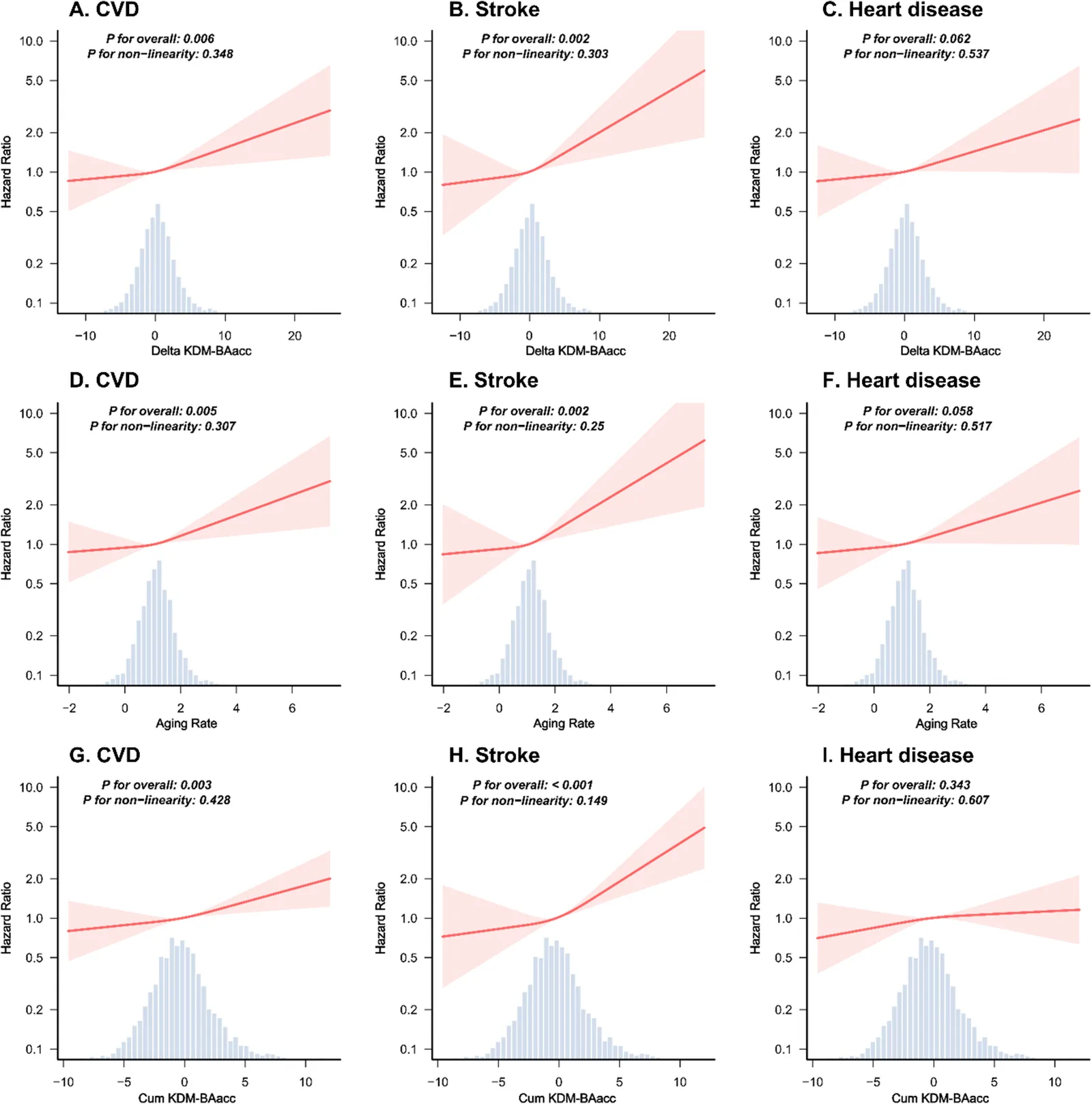
Dose-response relationships between dynamic biological aging indicators and the risk of incident cardiovascular diseases. RCS curves illustrate the associations of Delta KDM-BAacc (**A**–**C**), Aging Rate (**D**–**F**), and Cum KDM-BAacc (**G**–**I**) with the risk of incident CVD, stroke, and heart disease. The solid red lines represent the estimated HRs, and the shaded pink areas represent the 95% CIs. All models were adjusted for age, sex, BMI, marital status, residence, education level, smoking, drinking, hypertension, diabetes, dyslipidemia, and chronic kidney disease (Model 3). The blue histograms at the bottom of each plot show the density distribution of the respective aging indicators. P for overall and P for non-linearity are provided within each panel. Abbreviations: RCS, restricted cubic splines; KDM-BAacc, Klemera-Doubal method biological age acceleration; Cum, cumulative; CVD, cardiovascular disease; HR, hazard ratio; CI, confidence interval; BMI, body mass index

RCS analyses revealed significant linear dose-response relationships between all three dynamic aging indicators—Delta KDM-BAacc, Aging Rate, and Cum KDM-BAacc—and the risk of incident CVD and stroke (all P for overall < 0.01, P for non−linearity > 0.05), with the hazard ratios increasing monotonically as biological aging accelerated (Fig. 3). In contrast, the associations between these dynamic indicators and incident heart disease were either marginally significant or non-significant (P for overall > 0.05). These findings underscore that accelerated biological aging over time, whether characterized by absolute change, rate of progression, or cumulative burden, serves as a robust and linear predictor of future stroke and overall CVD events, independent of traditional cardiovascular risk factors including hypertension, diabetes, and dyslipidemia.

### Associations of annualized biological aging rate, change in age acceleration, cumulative aging burden, and extreme phenotypes with incident CVD

Table 3 details the associations between KDM-BA dynamic metrics and incident cardiovascular outcomes in the fully adjusted model (Model 3). Generally, dynamic acceleration of biological aging was significantly associated with an increased risk of composite CVD, stroke, and heart disease. Continuous increases in both Δ KDM-BAacc and Aging rate were robust predictors of all three endpoints. Specifically, each unit increase in Δ KDM-BAacc was associated with a 3% to 5% increase in the risk of composite CVD, stroke, and heart disease (*P* < 0.05 for all). When dichotomized, participants exhibiting accelerated aging (Δ KDM-BAacc ≥ 0) faced a significantly elevated hazard of composite CVD (HR:1.27, 95% CI: 1.11–1.45) and stroke (HR: 1.43, 95% CI: 1.15–1.77) compared to those with decelerated aging. Similarly, an Aging Rate ≥ 1 significantly conferred a 17% to 34% excess risk across all cardiovascular outcomes compared to a slower aging rate (< 1). The impact of cumulative aging load (Cum KDM-BAacc) and extreme phenotypes varied by outcome type. In Model 3, higher cumulative acceleration significantly predicted incident composite CVD and stroke, but the association with heart disease was attenuated and did not reach statistical significance (*P* = 0.173). Notably, participants in the highest tertile (T3) of cumulative burden had a 1.51-fold increased hazard of stroke compared to the lowest tertile (T1) (HR 1.51, 95% CI 1.14–2.01). This pattern was further corroborated by the extreme phenotype analysis. Compared to the “Anti-aging” group (sustained lowest quartile of acceleration), the “Rapid aging” group (sustained highest quartile) demonstrated a marked susceptibility to stroke (HR: 1.97, 95% CI: 1.23–3.17, *P* = 0.005) and composite CVD (HR: 1.33, 95% CI: 1.01–1.75, *P* = 0.043). However, no significant excess risk for heart disease was observed in the Rapid aging group after full adjustment (*P* = 0.452). Results from the minimally adjusted models (Models 1 and 2) are presented in table S3. The direction of associations remained consistent with the main findings, although effect sizes were generally larger prior to the inclusion of clinical covariates.

**Table 3:** Associations between KDM-BA dynamic metrics with the risk of CVD

Groups	CVD HR (95% CI)	Stroke HR (95% CI)	Heart disease HR (95% CI)
Δ KDM-BAacc			
Continuous (per unit)	1.03 (1.01 ~ 1.05) **	1.05 (1.02 ~ 1.09) **	1.03 (1.01 ~ 1.05) *
Categorical			
< 0 (decelerated)	1.00 (Reference)	1.00 (Reference)	1.00 (Reference)
≥ 0 (accelerated)	1.27 (1.11 ~ 1.45) ***	1.43 (1.15 ~ 1.77) **	1.22 (1.05 ~ 1.43) *
Aging rate			
Continuous (per unit)	1.13 (1.04 ~ 1.23) **	1.23 (1.08 ~ 1.40) **	1.11 (1.01 ~ 1.23) *
Categorical			
< 1 (Slow)	1.00 (Reference)	1.00 (Reference)	1.00 (Reference)
≥ 1 (Fast)	1.22 (1.07 ~ 1.39) **	1.34 (1.08 ~ 1.67) **	1.17 (1.01 ~ 1.37) *
Cumulative KDM-BAacc			
Continuous (per unit)	1.04 (1.02 ~ 1.07) **	1.10 (1.06 ~ 1.15) ***	1.02 (0.99 ~ 1.05)
Tertiles			
T1	1.00 (Reference)	1.00 (Reference)	1.00 (Reference)
T2	1.12 (0.94 ~ 1.34)	1.08 (0.80 ~ 1.45)	1.19 (0.97 ~ 1.47)
T3	1.25 (1.05 ~ 1.49) *	1.51 (1.14 ~ 2.01) **	1.18 (0.95 ~ 1.45)
Extreme phenotype			
Anti-aging	1.00 (Reference)	1.00 (Reference)	1.00 (Reference)
Rapid aging	1.33 (1.01 ~ 1.75) *	1.97 (1.23 ~ 3.17) **	1.13 (0.82 ~ 1.57)

*HR* hazard ratio, *CI* confidence interval, *CVD* cardiovascular disease, *CKD* chronic kidney disease, *BMI* body mass index, *KDM-BAacc* KDM biological age acceleration; Δ, delta (change); *Cum* cumulative. Models were adjusted for age, sex, residence, education level, marital status, smoking status, drinking status, BMI, hypertension, diabetes, dyslipidemia, and CKD. *p < 0.05; **p < 0.01; ***p < 0.001

### Subgroup analyses of biological aging reversal

To evaluate whether the cardioprotective benefits associated with reversing biological aging (accelerated to non-accelerated) relative to sustained acceleration were modified by participant characteristics, we performed stratified analyses (Fig. S1). The inverse association between the accelerated to non-accelerated trajectory and the risk of composite CVD outcomes remained broadly consistent across all strata, including age, sex, residence, BMI, and comorbidities. Notably, the reduction in stroke risk was particularly profound and uniform across subgroups. We observed no significant multiplicative interactions between the aging trajectory and any stratifying variables (all P for interaction > 0.05), indicating that the cardiovascular benefits of ameliorating biological aging are robust and not limited to specific subpopulations. For instance, the protective effect persisted regardless of smoking status or the presence of hypertension, suggesting an independent pathway of benefit. Subgroup analyses based on the K-means clustering trajectories (high to low vs. stable high) generally corroborated the findings from the primary analysis, demonstrating a robust protective trend against CVD and stroke across most strata (Fig. S2). However, unlike the primary definition, we identified a significant interaction by dyslipidemia status in the cluster-based model (P for interaction < 0.05 for both CVD and stroke). Specifically, the cardiovascular benefits of the decelerating aging trajectory (High to Low) were more pronounced in participants without dyslipidemia (CVD HR: 0.59; Stroke HR: 0.40) compared to those with dyslipidemia. No other significant interactions were detected, reinforcing the stability of the association across diverse demographic and clinical profiles.

### Sensitivity analyses

We performed several sensitivity analyses to verify the robustness of our findings, including using Fine-Gray competing risk models, multiple imputation for missing data, and excluding participants who developed outcomes during the first follow-up interval (Wave 4) to minimize reverse causality. The results were generally consistent with the main analysis (Tables S4–S9). Across all models, the “sustained non- accelerated " and " accelerated to non-accelerated " trajectories consistently showed a protective effect against CVD and stroke. Notably, the “Rapid aging” phenotype and high cumulative KDM-BAacc remained strong predictors of adverse outcomes. In the analysis excluding early events, the “Rapid aging” phenotype was associated with a more than six-fold increase in stroke risk (HR = 6.43, 95% CI: 1.83–22.53) and was significantly associated with heart disease (HR = 1.99, 95% CI: 1.05–3.77), highlighting the long-term impact of sustained biological aging.

## Discussion

In this nationally representative prospective cohort study, we provide a comprehensive characterization of the longitudinal dynamics of KDM biological age acceleration and its consequential impact on cardiovascular outcomes among middle-aged and older Chinese adults. Moving beyond static baseline assessments, our analysis reveals that biological aging is a fluid process exhibiting significant plasticity. A pivotal clinical observation from our investigation is that the acceleration of biological aging is not irreversible. Individuals who successfully transitioned from an accelerated to a decelerated aging state achieved a substantial reduction in CVD risk, particularly for stroke, with protective benefits comparable to those maintaining a consistently slow aging trajectory. Furthermore, we established a robust, linear dose-response relationship where both the velocity of physiological decline (aging rate) and the cumulative burden of accelerated aging served as independent predictors of adverse events. Notably, the predictive strength of these dynamic metrics was most profound for cerebrovascular incidents, with extreme phenotypes of sustained rapid aging conferring a drastic amplification in stroke risk. Collectively, these findings underscore the critical importance of monitoring longitudinal fluctuations in biological age and highlight the potential of ameliorating physiological decline as a viable strategy for cardiovascular prevention.

While a plethora of evidence from large-scale cohorts like the China Kadoorie Biobank and the UK Biobank has cemented the association between KDM biological age acceleration and the spectrum of cardiovascular-kidney-metabolic diseases [13, 14, 16, 25–27], these previous insights have been largely constrained by a static viewpoint. Previous scholarship has predominantly treated biological aging as a fixed baseline exposure, a perspective overlooking its temporal fluidity. By shifting the lens to a dynamic perspective within a longitudinal framework, our study unveils a critical nuance indicating that the trajectory of aging matters just as much as the baseline state. We observed that the cardiovascular advantage is strictly reserved for those maintaining a sustained decelerated aging profile. Crucially, a shift toward accelerated aging erases prior health benefits, elevating risks to levels seen in chronically accelerated individuals. Quantitatively, an upward drift in KDM-BAacc or a rapid aging rate relative to chronological time was associated with a 22–27% excess risk of composite CVD and a striking 34–43% increase in stroke susceptibility. Most alarmingly, individuals categorized as “Rapid agers” at both time points faced a near-doubling of stroke risk compared to their “Anti-aging” counterparts, highlighting the compounding danger of unmitigated physiological decline.

Perhaps the most clinically actionable insight from our analysis is the validation of biological age reversal as a viable therapeutic target. In stark contrast to the immutability of chronological time, we identified that 17.09% of our cohort achieved a reversion from an accelerated to a decelerated biological state, a shift translating into tangible reductions in stroke risk. This phenomenon echoes findings from the landmark CALERIE trial, where caloric restriction significantly blunted the annual increase in KDM age [28], and the TRIIM trial, which suggested pharmacological interventions could rewind the biological clock [29]. The mechanism linking this decelerated biological aging to preserved cardiovascular health is likely pleiotropic and deeply rooted in cellular bioenergetics. At the micro-level, this macroscopic biological U-turn is actively discussed in the scientific literature in the context of mitochondrial biosensorics and rejuvenation [19, 20]. As vital biosensors, mitochondria respond to systemic insults, and restoring their homeostasis effectively dampens chronic low-grade inflammation, a primary driver of atherosclerosis. Consequently, the mitigation of KDM acceleration serves as a composite proxy for this cellular rejuvenation, establishing biological age deceleration as a distinct pathway for cardiovascular protection.

The robust nature of these findings is grounded in several methodological strengths. To our knowledge, this is the premier longitudinal investigation to systematically characterize dynamic KDM-BA trajectories in a nationally representative Chinese cohort. We employed a comprehensive array of metrics, including categorical transitions, data-driven clustering, and cumulative burden, ensuring our findings are not merely statistical artifacts of a single definition. Furthermore, rigorous statistical modeling reinforces the reliability of the observed dose-response relationships.

### Limitations

Several limitations warrant consideration. The observational design precludes definitive causal inferences between aging dynamics and cardiovascular outcomes. Crucially, our findings derive exclusively from a Chinese population. Because biological aging trajectories are profoundly influenced by gene-environment interactions, dietary habits, and socioeconomic structures, the specific KDM-BAacc patterns observed here may not directly generalize to other ethnicities. Future studies must validate these dynamic metrics across multi-ethnic cohorts to establish globally applicable PPPM guidelines. Furthermore, while KDM-BA relies on validated routine clinical biomarkers, residual confounding from unmeasured factors cannot be entirely ruled out. Our future research will integrate multi-omics data, including epigenetics and metabolomics, to enhance the precision of biological age algorithms. Lastly, relying on self-reported physician diagnoses for CVD outcomes introduces potential recall bias, although this method is a validated standard in large epidemiological surveys like CHARLS.

### Expert recommendations and outlook in the framework of PPPM/3PM

The conventional reactive medical model predominantly intervenes only after the clinical manifestation of cardiovascular events. Clinical trials have repeatedly demonstrated the limitations of this delayed approach. For example, stable myocardial infarction survivors retain a high incidence of subsequent events despite standard therapies [30], and intensifying secondary prevention to optimize traditional risk factors often fails to significantly alter long-term prognosis [31]. These realities emphasize that **escalating care s**olely after disease onset is insufficient, underscoring an urgent need for a paradigm shift from reactive medical services to the holistic approach of PPPM. Rather than merely presenting epidemiological associations, our longitudinal findings provide a concrete, actionable framework for implementing this shift across three primary dimensions.

Moving beyond epidemiological associations, our longitudinal findings provide an actionable framework for implementing this shift. For routine medical application, we recommend integrating longitudinal KDM-BAacc profiling into annual primary care examinations. Utilizing standard and cost-effective clinical biomarkers allows physicians to calculate and track biological aging rates over time. This profiling functions as a predictive diagnostic tool to identify individuals in suboptimal health with reversible damage prior to disease onset. When patients exhibit rapid or accelerated aging trajectories, clinicians can immediately implement targeted preventive measures including intensive lifestyle modifications and early pharmacological interventions. This strategy personalizes medical services based on individual aging trajectories and decisively surpasses conventional generalized clinical approaches.

### Predictive diagnostics

Although individuals of identical chronological age frequently exhibit varying degrees of biological senescence, traditional guidelines still rely heavily on immutable demographic metrics [32]. Historically, accelerated biological aging has been recognized as an indicator of rapid physiological deterioration, reflecting significant systemic dysfunction and correlating with elevated morbidity and mortality [16, 33]. However, a critical nuance has been largely overlooked in previous literature: patients presenting with the exact same baseline biological age acceleration may experience vastly divergent aging trajectories over time. Our investigation pioneers the use of longitudinal KDM-BAacc fluctuations as a highly precise, forward-looking predictive instrument. Many individuals exist in suboptimal health conditions, harboring reversible damage long before overt stroke or myocardial infarction occurs [6]. By monitoring the velocity of biological aging, clinicians can effectively stratify cardiovascular risk among individuals who appear clinically identical at a single cross-section. Looking toward the future, while systemic blood biomarkers remain the current epidemiological standard, the integration of innovative predictive diagnostic technologies, such as tear fluid multi-omics and continuous digital health monitoring [8], will further revolutionize our ability to capture these dynamic aging trajectories in real-time.

### Targeted prevention

A critical limitation of the conventional reactive medical model is its failure to capitalize on the reversible window of physiological decline. Addressing whether ameliorating age acceleration during this critical window translates to tangible cardiovascular risk reduction represents a paramount clinical contribution of our investigation. By tracking the longitudinal dynamics of KDM-BAacc, we empirically demonstrated that individuals successfully transitioning from an accelerated to a non-accelerated aging state profoundly attenuated their stroke susceptibility. This dynamic shift effectively restored their cardiovascular risk profile to a level comparable to those maintaining a consistently slow aging trajectory, robustly confirming that **physiological deterioration is a highly **modifiable process rather than an inevitable fate.

How can healthcare systems effectively operationalize this biological reversibility? Translating this epidemiological observation into clinical practice requires moving beyond generic lifestyle advice to concrete implementation strategies targeting the cellular roots of aging. As mitochondria act as central hubs for metabolic regulation, targeted interventions restoring their bioenergetic efficiency can effectively rewind the biological clock and mitigate the systemic insults driving atherosclerosis [20]. Consequently, targeted prevention must focus on specific biological processes including genomic instability, telomere attrition, epigenetic alterations, and mitochondrial dysfunction [34]. For instance, supplementing with specific anti-aging compounds and senolytics offers a promising avenue to reduce cardiovascular risk and extend healthspan. Polyphenols, such as grape skin extracts and citrus flavanones, alongside curcumin, exert profound anti-inflammatory effects and regulate the senescence-associated secretory phenotype while enhancing autophagy [35, 36]. Furthermore, restoring cellular energy dynamics is crucial. Supplementing with NAD+ precursors like nicotinamide riboside or NMN activates the SIRT1 pathway, whereas agents like coenzyme Q10, resveratrol, and quercetin directly improve oxidative phosphorylation efficiency and ATP generation [36]. Research indicates that enhancing NAD+ levels can ameliorate telomere attrition, a core feature of aging, and improve lipid profiles in older adults [37, 38]. Additionally, for patients exhibiting aging driven by gut dysbiosis, targeted prebiotic supplementation holds the potential to delay systemic decline [39]. By implementing these targeted strategies in primary care settings, healthcare providers can offer individualised protection against the health-to-disease transition. This proactive approach empowers clinicians to halt disease progression at its root, fundamentally shifting the paradigm from managing the devastating aftermath of a stroke to preserving long-term cardiovascular resilience.

### Personalisation of medical services

Assuming a uniform aging process across populations is a fundamental flaw in traditional disease management. Our data-driven clustering reveals profound inter-individual heterogeneity in aging trajectories, suggesting that **categorizing patients into distinct **dynamic phenotypes enables the delivery of highly individualized care. This concept is particularly vital in community-based chronic disease management, where evaluating dynamic changes in KDM-BAacc helps healthcare workers adjust intervention intensity. For example, recent evidence indicates that biological age acceleration significantly mediates the association between various obesity indices and cardiovascular disease, with this mediating effect reaching up to 25.46% and being particularly pronounced among middle-aged populations [40]. Therefore, if a patient’s trajectory shifts from a stable non-accelerated state to an accelerated one, signaling an impending increase in stroke risk, physicians must immediately intensify the intervention. This could involve stricter management of blood pressure and glucose alongside the initiation of mitochondrial-targeted anti-aging therapies.

Beyond tracking overall trajectories, interventions must align with the specific pathophysiological drivers of a patient’s aging phenotype. Our subgroup analyses indicate that younger individuals and those without baseline metabolic disorders derive the most pronounced cardiovascular benefits from decelerating their biological aging, highlighting these demographics as prime candidates for early proactive measures. Furthermore, for patients categorized in the “Low to high” aging cluster, identifying the primary driving factors is essential. For instance, research demonstrates that low vitamin D combined with elevated C-reactive protein significantly exacerbates KDM-BAacc [41]. For such patients, the therapeutic focus should lean heavily toward targeted anti-inflammatory strategies. If the acceleration is predominantly driven by metabolic abnormalities, the priority shifts to aggressive metabolic control and ameliorating insulin resistance [40]. This precise matching of therapeutic modalities to a patient’s unique biological aging phenotype, thereby moving away from a conventional one-size-fits-all approach, embodies the core essence of personalised medicine.

### The paradigm shifts from reactive to PPPM/3PM and go beyond the state of the art

The current state of the art in cardiovascular management relies heavily on reactive interventions initiated only after irreversible vascular damage has occurred, inherently treating physiological aging as a uniform and inevitable decline. How can modern healthcare transcend these critical limitations? Our investigation provides a robust epidemiological foundation to drive a paradigm shift toward predictive, preventive, and personalised medicine by demonstrating that biological aging is actually a highly dynamic and reversible process. By utilizing longitudinal KDM-BAacc fluctuations as a forward-looking predictive instrument, clinicians can identify patients trapped in suboptimal health conditions long before overt clinical symptoms emerge. Capturing this precise temporal window where physiological damage remains fully reversible naturally dictates a profound transformation in clinical implementation. Rather than prescribing generic lifestyle modifications, physicians can deploy targeted interventions addressing the cellular roots of senescence, such as optimizing mitochondrial bioenergetics, the moment a patient shifts toward a rapid aging trajectory. This proactive approach is further refined by our data-driven clustering, which exposes the fundamental flaw of one-size-fits-all treatment algorithms. By matching therapeutic modalities to a patient’s unique biological aging phenotype, ranging from targeted anti-inflammatory regimens for C-reactive protein-driven acceleration to aggressive metabolic control for obesity-driven cases, healthcare providers can maximize therapeutic efficacy. Ultimately, integrating these dynamic biological assessments transforms cardiovascular medicine from a delayed rescue operation into a proactive, individualized protection plan, firmly establishing a new standard of care within the 3PM framework.

## Supplementary Information

Below is the link to the electronic supplementary material.


Supplementary Material 1



Supplementary Material 2


## Data Availability

The datasets used in this study are publicly available on the CHARLS website: [https://charls.pku.edu.cn/].

## Abbreviations

ANOVA: Analysis of variance
BA: Biological age
BAacc: Biological age acceleration
BMI: Body mass index
BUN: Blood urea nitrogen
CHARLS: China Health and Retirement Longitudinal Study
CI: Confidence interval
CKD: Chronic kidney disease
CRP: C-reactive protein
CVD: Cardiovascular disease
eGFR: Estimated glomerular filtration rate
HbA1c: Glycated hemoglobin
HDL-C: High-density lipoprotein cholesterol
HR: Hazard ratio
IQR: Interquartile range
KDM: Klemera-Doubal method
LDL-C: Low-density lipoprotein cholesterol
PLT: Platelet count
PPPM / 3PM: Predictive, Preventive, and Personalised Medicine
RCS: Restricted cubic splines
SBP: Systolic blood pressure
SD: Standard deviation
TC: Total cholesterol
TG: Triglycerides
