# Supplementary material for: Dynamic reversibility of biological aging and risk of cardiovascular disease and stroke: a longitudinal cohort study
Source: EPMA J. 2026 Jul 2;17(3):501–15. doi: 10.1007/s13167-026-00460-9 (PMC13530061; doi:10.1007/s13167-026-00460-9)
Supplement: Supplementary file 1 [file 13167_2026_460_MOESM1_ESM.docx]

**Calculation of KDM-BA**

The KDM biological age (KDM-BA) for each participant was calculated based on the Klemera-Doubal method, a supervised machine learning algorithm that estimates biological age by minimizing the distance between biological markers and chronological age. The algorithm synthesizes m biomarkers into a single biological age estimate using the following equation:

$$KDM-BA_{j}=\frac{\sum_{i=1}^{m} \left( \frac{x_{ji}-q_{i}}{k_{i}} \right)\left( \frac{k_{i}}{s_{i}} \right)^{2}+\frac{CA_{j}}{s_{BA}^{2}}}{\sum_{i=1}^{m} \left( \frac{k_{i}}{s_{i}} \right)^{2}+\frac{1}{s_{BA}^{2}}}$$

KDM-BA*_j_* represents the estimated KDM-BA for individual *j*.

*x_ji_* is the observed value of biomarker *i* for individual *j*.

*CA_j_* is the chronological age of individual *j*.

*k_i_*, *q_i_*, and *s_i_* denote the slope, intercept, and root mean squared error (RMSE), respectively, derived from the linear regression of biomarker *i* on chronological age in the baseline training set.

*s*^2^*_BA_* is the variance of chronological age in the training sample.

*m* is the total number of biomarkers included in the algorithm (*m* = 8).

The term (*x_ji_*−*q_i_*)/*k_i_* represents the biological age predicted solely by biomarker *i*, while (*k_i_*/*s_i_*)^2^ serves as the weighting factor, assigning greater weight to biomarkers that are more strongly correlated with chronological age (larger slope *k*) and have less noise (smaller error *s*).

**Calculation of Biological Age Acceleration**

To quantify the deviation between an individual's biological and chronological age, we calculated the KDM biological age acceleration (KDM-BAacc) as follows:

$$KDM\text{-}BA_{acc}=KDM-BA-CA$$

A positive KDM-BAacc indicates that an individual is aging faster than expected for their chronological age (accelerated aging), whereas a negative value suggests a slower aging process (decelerated aging). This metric was calculated for each wave and subsequently used to derive dynamic change variables (e.g., ΔKDM-BAacc, Aging rate, and Cumulative KDM-BAacc).

**Table S1** Baseline characteristics of participants stratified by K-means clustering of biological aging trajectories

| Characteristics | Total  (N = 4756) | Stable high  (N = 685) | Low to high (N = 991) | Stable low (N = 1352) | High to low (N = 1728) | P |
| --- | --- | --- | --- | --- | --- | --- |
| Age, years, mean ± SD | 58.55 ± 8.74 | 59.12 ± 8.72 | 58.45 ± 9.00 | 58.58 ± 8.75 | 58.35 ± 8.58 | 0.257 |
| Sex, n (%) |  |  |  |  |  | < 0.001 |
| Female | 2517 (52.9) | 474 (69.2) | 523 (52.8) | 837 (61.9) | 683 (39.5) |  |
| Male | 2239 (47.1) | 211 (30.8) | 468 (47.2) | 515 (38.1) | 1045 (60.5) |  |
| Residence, n (%) |  |  |  |  |  | 0.018 |
| Urban | 1494 (31.4) | 245 (35.8) | 290 (29.3) | 404 (29.9) | 555 (32.1) |  |
| Rural | 3262 (68.6) | 440 (64.2) | 701 (70.7) | 948 (70.1) | 1173 (67.9) |  |
| Education level, n (%) |  |  |  |  |  | < 0.001 |
| < High school | 4379 (92.1) | 652 (95.2) | 927 (93.5) | 1239 (91.6) | 1561 (90.3) |  |
| ≥ High school | 377 (7.9) | 33 (4.8) | 64 (6.5) | 113 (8.4) | 167 (9.7) |  |
| Marital status, n (%) |  |  |  |  |  | 0.305 |
| Married/cohabiting | 4087 (85.9) | 581 (84.8) | 869 (87.7) | 1154 (85.4) | 1483 (85.8) |  |
| Other | 669 (14.1) | 104 (15.2) | 122 (12.3) | 198 (14.6) | 245 (14.2) |  |
| Smoking status, n (%) |  |  |  |  |  | < 0.001 |
| No | 3261 (68.8) | 530 (77.7) | 701 (71.0) | 1002 (74.4) | 1028 (59.7) |  |
| Yes | 1479 (31.2) | 152 (22.3) | 287 (29.0) | 345 (25.6) | 695 (40.3) |  |
| Drinking status, n (%) |  |  |  |  |  | < 0.001 |
| No | 3098 (65.2) | 521 (76.1) | 637 (64.3) | 917 (67.9) | 1023 (59.3) |  |
| Yes | 1654 (34.8) | 164 (23.9) | 353 (35.7) | 434 (32.1) | 703 (40.7) |  |
| BMI, kg/m², mean ± SD | 23.44 ± 3.77 | 24.94 ± 4.22 | 23.58 ± 3.85 | 22.73 ± 3.57 | 23.32 ± 3.52 | < 0.001 |
| BMI Categories, n (%) |  |  |  |  |  | < 0.001 |
| < 24 kg/m² | 2877 (61.1) | 299 (43.9) | 580 (59.2) | 931 (69.7) | 1067 (62.2) |  |
| ≥ 24 kg/m² | 1835 (38.9) | 382 (56.1) | 400 (40.8) | 405 (30.3) | 648 (37.8) |  |
| SBP, mmHg, mean ± SD | 127.74 ± 20.42 | 147.13 ± 22.24 | 126.31 ± 17.01 | 116.31 ± 14.66 | 129.83 ± 19.00 | < 0.001 |
| Hypertension, n (%) | 2141 (46.0) | 556 (81.8) | 457 (47.1) | 279 (21.3) | 849 (50.2) | < 0.001 |
| Diabetes, n (%) | 666 (14.0) | 167 (24.4) | 139 (14.0) | 134 (9.9) | 226 (13.1) | < 0.001 |
| Dyslipidemia, n (%) | 2223 (46.7) | 396 (57.8) | 474 (47.8) | 561 (41.5) | 792 (45.8) | < 0.001 |
| CKD, n (%) | 315 (6.6) | 77 (11.2) | 57 (5.8) | 67 (5.0) | 114 (6.6) | < 0.001 |
| TC, mg/dL | 193.04 ± 37.72 | 207.62 ± 42.06 | 191.15 ± 35.15 | 186.85 ± 35.24 | 193.18 ± 37.69 | < 0.001 |
| TG, mg/dL, median (IQR) | 103.54 (73.5, 151.3) | 118.59 (82.3, 182.3) | 106.20 (77.0, 155.8) | 104.43 (74.3, 148.0) | 97.35 (70.8, 141.8) | < 0.001 |
| HDL-C, mg/dL | 51.41 ± 15.27 | 49.91 ± 14.96 | 51.28 ± 15.32 | 51.12 ± 15.00 | 52.31 ± 15.51 | 0.004 |
| HbA1c, % | 5.25 ± 0.80 | 5.58 ± 1.29 | 5.22 ± 0.68 | 5.13 ± 0.60 | 5.24 ± 0.70 | < 0.001 |
| Creatinine, mg/dL | 0.76 ± 0.17 | 0.85 ± 0.22 | 0.75 ± 0.16 | 0.68 ± 0.14 | 0.80 ± 0.16 | < 0.001 |
| BUN, mg/dL | 15.65 ± 4.29 | 18.10 ± 5.20 | 15.44 ± 3.81 | 13.66 ± 3.28 | 16.37 ± 4.15 | < 0.001 |
| eGFR, mL/min/1.73m² | 97.32 ± 12.96 | 87.55 ± 16.41 | 98.32 ± 11.71 | 102.46 ± 9.58 | 96.60 ± 12.06 | < 0.001 |
| CRP, mg/L, median (IQR) | 0.96 (0.53, 1.96) | 1.62 (0.87, 3.20) | 0.92 (0.53, 1.76) | 0.63 (0.41, 1.17) | 1.15 (0.60, 2.41) | < 0.001 |
| Platelets, 10⁹/L | 211.27 ± 71.60 | 202.76 ± 69.01 | 213.72 ± 74.43 | 220.95 ± 73.61 | 205.66 ± 68.40 | < 0.001 |
| KDM-BA, years | 58.40 ± 9.24 | 63.47 ± 9.03 | 57.90 ± 9.07 | 55.38 ± 8.82 | 59.05 ± 8.76 | < 0.001 |
| KDM-BAacc, median (IQR) | -0.26 (-2.00, 1.53) | 3.92 (2.82, 5.42) | -0.37 (-1.46, 0.58) | -2.89 (-4.05, -2.03) | 0.54 (-0.43, 1.63) | < 0.001 |
| Aging rate, median (IQR) | 1.06 (0.64, 1.47) | 0.91 (0.45, 1.40) | 1.63 (1.39, 2.05) | 1.14 (0.80, 1.51) | 0.73 (0.40, 1.00) | < 0.001 |
| Δ KDM-BAacc, median (IQR) | -0.10 (-1.76, 1.57) | -0.69 (-2.50, 1.26) | 2.18 (1.24, 3.87) | 0.23 (-1.11, 1.73) | -1.39 (-2.73, -0.32) | < 0.001 |
| Cum KDM-BAacc, median (IQR) | -0.26 (-1.80, 1.31) | 3.58 (2.78, 5.01) | 0.91 (0.11, 1.69) | -2.73 (-3.75, -2.04) | -0.26 (-0.93, 0.58) | < 0.001 |
| Heart disease, n (%) | 667 (14.0) | 121 (17.7) | 165 (16.6) | 168 (12.4) | 213 (12.3) | < 0.001 |
| Stroke, n (%) | 352 (7.4) | 82 (12.0) | 91 (9.2) | 77 (5.7) | 102 (5.9) | < 0.001 |
| CVD, n (%) | 931 (19.6) | 185 (27.0) | 227 (22.9) | 229 (16.9) | 290 (16.8) | < 0.001 |

Abbreviations: BMI, body mass index; SBP, systolic blood pressure; CKD, chronic kidney disease; TC, total cholesterol; TG, triglycerides; HDL-C, high-density lipoprotein cholesterol; HbA1c, glycated hemoglobin; BUN, blood urea nitrogen; eGFR, estimated glomerular filtration rate; CRP, C-reactive protein; KDM-BA, Klemera-Doubal method biological age; KDM-BAacc, KDM biological age acceleration; Cum KDM-BAacc, cumulative KDM biological age acceleration; CVD, cardiovascular disease; SD, standard deviation; IQR, interquartile range. Data are presented as mean ± SD for normally distributed continuous variables, median (IQR) for skewed continuous variables, and n (%) for categorical variables. P-values were derived from one-way ANOVA or Kruskal-Wallis tests for continuous variables and Chi-square tests for categorical variables. P values < 0.05 are indicated in bold.

**Table S2** Associations of biological aging trajectories and clusters with the risk of CVD

| Outcomes and groups | Model1 | | Model2 | | Model3 | |
| --- | --- | --- | --- | --- | --- | --- |
|  | HR (95%CI) | *P* | HR (95%CI) | *P* | HR (95%CI) | *P* |
| Incident CVD |  |  |  |  |  |  |
| Trajectory groups |  |  |  |  |  |  |
| Sustained acc | 1.00 (Reference) |  | 1.00 (Reference) |  | 1.00 (Reference) |  |
| Sustained non-acc | 0.62 (0.53 ~ 0.72) | < 0.001 | 0.66 (0.56 ~ 0.77) | < 0.001 | 0.77 (0.65 ~ 0.92) | 0.003 |
| Non-acc to acc | 0.86 (0.71 ~ 1.04) | 0.125 | 0.87 (0.72 ~ 1.06) | 0.172 | 0.98 (0.80 ~ 1.21) | 0.875 |
| Acc to non-acc | 0.68 (0.56 ~ 0.83) | < 0.001 | 0.69 (0.57 ~ 0.85) | < 0.001 | 0.72 (0.59 ~ 0.89) | 0.002 |
| Cluster groups |  |  |  |  |  |  |
| Stable high | 1.00 (Reference) |  | 1.00 (Reference) |  | 1.00 (Reference) |  |
| Stable low | 0.57 (0.47 ~ 0.69) | < 0.001 | 0.62 (0.51 ~ 0.76) | < 0.001 | 0.77 (0.62 ~ 0.96) | 0.022 |
| Low to high | 0.83 (0.68 ~ 1.01) | 0.066 | 0.86 (0.71 ~ 1.05) | 0.148 | 0.98 (0.80 ~ 1.21) | 0.860 |
| High to low | 0.62 (0.51 ~ 0.75) | < 0.001 | 0.65 (0.54 ~ 0.79) | < 0.001 | 0.73 (0.60 ~ 0.89) | 0.002 |
| Incident Stroke |  |  |  |  |  |  |
| Trajectory groups |  |  |  |  |  |  |
| Sustained Acc | 1.00 (Reference) |  | 1.00 (Reference) |  | 1.00 (Reference) |  |
| Sustained non-acc | 0.50 (0.39 ~ 0.64) | < 0.001 | 0.55 (0.42 ~ 0.71) | < 0.001 | 0.63 (0.47 ~ 0.82) | < 0.001 |
| Non-acc to acc | 0.76 (0.56 ~ 1.03) | 0.080 | 0.78 (0.58 ~ 1.07) | 0.123 | 0.85 (0.62 ~ 1.18) | 0.337 |
| Acc to non-acc | 0.43 (0.30 ~ 0.62) | < 0.001 | 0.44 (0.31 ~ 0.63) | < 0.001 | 0.46 (0.32 ~ 0.65) | < 0.001 |
| Cluster Groups |  |  |  |  |  |  |
| Stable high | 1.00 (Reference) |  | 1.00 (Reference) |  | 1.00 (Reference) |  |
| Stable low | 0.43 (0.31 ~ 0.59) | < 0.001 | 0.48 (0.35 ~ 0.66) | < 0.001 | 0.58 (0.41 ~ 0.82) | 0.002 |
| Low to high | 0.73 (0.54 ~ 0.98) | 0.038 | 0.75 (0.55 ~ 1.02) | 0.066 | 0.83 (0.60 ~ 1.14) | 0.245 |
| High to low | 0.46 (0.34 ~ 0.61) | < 0.001 | 0.48 (0.36 ~ 0.65) | < 0.001 | 0.53 (0.39 ~ 0.73) | < 0.001 |
| Incident heart disease |  |  |  |  |  |  |
| Trajectory groups |  |  |  |  |  |  |
| Sustained Acc | 1.00 (Reference) |  | 1.00 (Reference) |  | 1.00 (Reference) |  |
| Sustained non-acc | 0.69 (0.57 ~ 0.83) | < 0.001 | 0.73 (0.60 ~ 0.88) | 0.001 | 0.86 (0.70 ~ 1.06) | 0.148 |
| Non-Acc to Acc | 1.00 (0.79 ~ 1.25) | 0.972 | 1.01 (0.80 ~ 1.27) | 0.938 | 1.16 (0.91 ~ 1.47) | 0.227 |
| Acc to non-acc | 0.84 (0.67 ~ 1.06) | 0.138 | 0.85 (0.67 ~ 1.07) | 0.171 | 0.89 (0.70 ~ 1.12) | 0.324 |
| Cluster Groups |  |  |  |  |  |  |
| Stable high | 1.00 (Reference) |  | 1.00 (Reference) |  | 1.00 (Reference) |  |
| Stable low | 0.65 (0.52 ~ 0.83) | < 0.001 | 0.71 (0.56 ~ 0.90) | 0.005 | 0.89 (0.68 ~ 1.15) | 0.372 |
| Low to high | 0.96 (0.76 ~ 1.21) | 0.721 | 1.00 (0.79 ~ 1.27) | 0.994 | 1.15 (0.90 ~ 1.47) | 0.272 |
| High to low | 0.75 (0.59 ~ 0.94) | 0.012 | 0.78 (0.62 ~ 0.98) | 0.033 | 0.88 (0.69 ~ 1.11) | 0.273 |

Abbreviations: HR, hazard ratio; CI, confidence interval; CVD, cardiovascular disease; Acc, accelerated; Non-acc, non-accelerated. Model 1: Adjusted for age, sex, marital status, residence, and education level. Model 2: Adjusted for covariates in Model 1 plus BMI, smoking status, and drinking status. Model 3: Adjusted for covariates in Model 2 plus hypertension, diabetes, dyslipidemia, and chronic kidney disease. Bold values indicate statistical significance (P < 0.05).

**Table S3 Associations between KDM-BA dynamic metrics with** the risk of CVD

| Outcomes and groups | Model1 | | Model2 | | Model3 | |
| --- | --- | --- | --- | --- | --- | --- |
|  | HR (95%CI) | *P* | HR (95%CI) | *P* | HR (95%CI) | *P* |
| Incident CVD |  |  |  |  |  |  |
| Δ KDM-BAacc |  |  |  |  |  |  |
| Continuous (per unit) | 1.02 (1.00 ~ 1.04) | 0.065 | 1.02 (1.01 ~ 1.04) | 0.048 | 1.03 (1.01 ~ 1.05) | 0.004 |
| Categorical |  |  |  |  |  |  |
| < 0 (decelerated) | 1.00 (Reference) |  | 1.00 (Reference) |  | 1.00 (Reference) |  |
| ≥ 0 (accelerated) | 1.17 (1.03 ~ 1.33) | 0.015 | 1.18 (1.04 ~ 1.35) | 0.011 | 1.27 (1.11 ~ 1.45) | < 0.001 |
| Aging rate |  |  |  |  |  |  |
| Continuous (per unit) | 1.08 (1.00 ~ 1.17) | 0.064 | 1.09 (1.01 ~ 1.18) | 0.048 | 1.13 (1.04 ~ 1.23) | 0.004 |
| Categorical |  |  |  |  |  |  |
| < 1 (Slow) | 1.00 (Reference) |  | 1.00 (Reference) |  | 1.00 (Reference) |  |
| ≥ 1 (Fast) | 1.12 (0.98 ~ 1.27) | 0.095 | 1.13 (0.99 ~ 1.29) | 0.065 | 1.22 (1.07 ~ 1.39) | 0.003 |
| Cumulative KDM-BAacc |  |  |  |  |  |  |
| Continuous (per unit) | 1.08 (1.06 ~ 1.11) | < 0.001 | 1.07 (1.05 ~ 1.10) | < 0.001 | 1.04 (1.02 ~ 1.07) | 0.001 |
| Tertiles |  |  |  |  |  |  |
| T1 | 1.00 (Reference) |  | 1.00 (Reference) |  | 1.00 (Reference) |  |
| T2 | 1.25 (1.06 ~ 1.48) | 0.010 | 1.21 (1.02 ~ 1.44) | 0.027 | 1.12 (0.94 ~ 1.34) | 0.190 |
| T3 | 1.58 (1.35 ~ 1.85) | < 0.001 | 1.48 (1.26 ~ 1.74) | < 0.001 | 1.25 (1.05 ~ 1.49) | 0.012 |
| Extreme phenotype |  |  |  |  |  |  |
| Anti-aging | 1.00 (Reference) |  | 1.00 (Reference) |  | 1.00 (Reference) |  |
| **Rapid** aging | 1.90 (1.48 ~ 2.44) | < 0.001 | 1.71 (1.32 ~ 2.22) | < 0.001 | 1.33 (1.01 ~ 1.75) | 0.043 |
| Incident stroke |  |  |  |  |  |  |
| Δ KDM-BAacc |  |  |  |  |  |  |
| Continuous (per unit) | 1.04 (1.01 ~ 1.08) | 0.013 | 1.05 (1.01 ~ 1.08) | 0.008 | 1.05 (1.02 ~ 1.09) | 0.002 |
| Categorical |  |  |  |  |  |  |
| < 0 (decelerated) | 1.00 (Reference) |  | 1.00 (Reference) |  | 1.00 (Reference) |  |
| ≥ 0 (accelerated) | 1.30 (1.05 ~ 1.60) | 0.016 | 1.32 (1.07 ~ 1.63) | 0.011 | 1.43 (1.15 ~ 1.77) | 0.001 |
| Aging rate |  |  |  |  |  |  |
| Continuous (per unit) | 1.18 (1.03 ~ 1.35) | 0.014 | 1.19 (1.05 ~ 1.36) | 0.009 | 1.23 (1.08 ~ 1.40) | 0.002 |
| Categorical |  |  |  |  |  |  |
| < 1 (Slow) | 1.00 (Reference) |  | 1.00 (Reference) |  | 1.00 (Reference) |  |
| ≥ 1 (Fast) | 1.21 (0.98 ~ 1.50) | 0.079 | 1.23 (1.00 ~ 1.53) | 0.055 | 1.34 (1.08 ~ 1.67) | 0.008 |
| Cumulative KDM-BAacc |  |  |  |  |  |  |
| Continuous (per unit) | 1.14 (1.09 ~ 1.18) | < 0.001 | 1.12 (1.08 ~ 1.16) | < 0.001 | 1.10 (1.06 ~ 1.15) | < 0.001 |
| Tertiles |  |  |  |  |  |  |
| T1 | 1.00 (Reference) |  | 1.00 (Reference) |  | 1.00 (Reference) |  |
| T2 | 1.20 (0.90 ~ 1.60) | 0.215 | 1.13 (0.85 ~ 1.51) | 0.393 | 1.08 (0.80 ~ 1.45) | 0.611 |
| T3 | 1.88 (1.45 ~ 2.44) | < 0.001 | 1.73 (1.33 ~ 2.26) | < 0.001 | 1.51 (1.14 ~ 2.01) | 0.005 |
| Extreme phenotype |  |  |  |  |  |  |
| Anti-aging | 1.00 (Reference) |  | 1.00 (Reference) |  | 1.00 (Reference) |  |
| **Rapid** aging | 2.84 (1.82 ~ 4.41) | < 0.001 | 2.42 (1.55 ~ 3.79) | < 0.001 | 1.97 (1.23 ~ 3.17) | 0.005 |
| Incident heart disease |  |  |  |  |  |  |
| Δ KDM-BAacc |  |  |  |  |  |  |
| Continuous (per unit) | 1.02 (0.99 ~ 1.04) | 0.192 | 1.02 (0.99 ~ 1.04) | 0.156 | 1.03 (1.01 ~ 1.05) | 0.030 |
| Categorical |  |  |  |  |  |  |
| < 0 (decelerated) | 1.00 (Reference) |  | 1.00 (Reference) |  | 1.00 (Reference) |  |
| ≥ 0 (accelerated) | 1.14 (0.98 ~ 1.33) | 0.091 | 1.15 (0.99 ~ 1.34) | 0.072 | 1.22 (1.05 ~ 1.43) | 0.011 |
| Aging rate |  |  |  |  |  |  |
| Continuous (per unit) | 1.07 (0.97 ~ 1.17) | 0.187 | 1.07 (0.97 ~ 1.18) | 0.152 | 1.11 (1.01 ~ 1.23) | 0.029 |
| Categorical |  |  |  |  |  |  |
| < 1 (Slow) | 1.00 (Reference) |  | 1.00 (Reference) |  | 1.00 (Reference) |  |
| ≥ 1 (Fast) | 1.08 (0.93 ~ 1.26) | 0.305 | 1.10 (0.94 ~ 1.28) | 0.234 | 1.17 (1.01 ~ 1.37) | 0.046 |
| Cumulative KDM-BAacc |  |  |  |  |  |  |
| Continuous (per unit) | 1.06 (1.03 ~ 1.09) | < 0.001 | 1.05 (1.02 ~ 1.08) | < 0.001 | 1.02 (0.99 ~ 1.05) | 0.173 |
| Tertiles |  |  |  |  |  |  |
| T1 | 1.00 (Reference) |  | 1.00 (Reference) |  | 1.00 (Reference) |  |
| T2 | 1.32 (1.08 ~ 1.61) | 0.006 | 1.30 (1.06 ~ 1.59) | 0.010 | 1.19 (0.97 ~ 1.47) | 0.093 |
| T3 | 1.47 (1.22 ~ 1.77) | < 0.001 | 1.39 (1.14 ~ 1.68) | < 0.001 | 1.18 (0.95 ~ 1.45) | 0.130 |
| Extreme phenotype |  |  |  |  |  |  |
| Anti-aging | 1.00 (Reference) |  | 1.00 (Reference) |  | 1.00 (Reference) |  |
| **Rapid** aging | 1.60 (1.19 ~ 2.15) | 0.002 | 1.47 (1.09 ~ 2.00) | 0.013 | 1.13 (0.82 ~ 1.57) | 0.452 |

Abbreviations: HR, hazard ratio; CI, confidence interval; CVD, cardiovascular disease. Notes: Trajectory definitions: Persistent Acceleration (Sustained Acc); Resilience (Sustained Non-Acc); Worsening (Non-Acc to Acc); Reversed (Acc to Non-Acc). Model 1: Adjusted for age, sex, marital status, residence, and education level. Model 2: Adjusted for covariates in Model 1 plus BMI, smoking status, and drinking status. Model 3: Adjusted for covariates in Model 2 plus hypertension, diabetes, dyslipidemia, and chronic kidney disease. Bold values indicate statistical significance (P < 0.05).

**Fig. S1** Subgroup analyses of the associations between biological aging reversal, sustained acceleration, and risk of incident cardiovascular disease. Abbreviations: HR, hazard ratio; CI, confidence interval; BMI, body mass index; CKD, chronic kidney disease; CVD, cardiovascular disease. Models were adjusted for age, sex, residence, education, marital status, smoking, drinking, BMI, hypertension, diabetes, dyslipidemia, and CKD (covariates were excluded from the model when used as stratification variables). P for interaction was calculated using the likelihood ratio test by adding a multiplicative interaction term to the model. Comparison: Accelerated to non-accelerated vs. sustained accelerated.

**Fig. S2** Subgroup analyses of the associations between biological aging reversal, sustained acceleration, and risk of incident cardiovascular disease. Abbreviations: HR, hazard ratio; CI, confidence interval; BMI, body mass index; CKD, chronic kidney disease; CVD, cardiovascular disease. Models were adjusted for age, sex, residence, education, marital status, smoking, drinking, BMI, hypertension, diabetes, dyslipidemia, and CKD (covariates were excluded from the model when used as stratification variables). P for interaction was calculated using the likelihood ratio test by adding a multiplicative interaction term to the model. Comparison: High to low vs. stable high.

**Table S4** Sensitivity analysis: Associations of biological aging trajectories and clusters with the risk of incident CVD, stroke, and heart disease using Fine-Gray competing risk models

| Variables | CVD |  | Stroke |  | Heart disease |  |
| --- | --- | --- | --- | --- | --- | --- |
|  | SHR (95%CI) | *P* | SHR (95%CI) | *P* | SHR (95%CI) | *P* |
| Longitudinal trajectories |  |  |  |  |  |  |
| Sustained acc | 1.00 (Reference) |  | 1.00 (Reference) |  | 1.00 (Reference) |  |
| Sustained non-acc | 0.79 (0.67 ~ 0.94) | 0.007 | 0.65 (0.49 ~ 0.86) | 0.002 | 0.88 (0.72 ~ 1.08) | 0.213 |
| Non-acc to acc | 0.97 (0.80 ~ 1.18) | 0.797 | 0.85 (0.61 ~ 1.16) | 0.302 | 1.14 (0.91 ~ 1.44) | 0.252 |
| Acc to non-acc | 0.73 (0.60 ~ 0.90) | 0.002 | 0.46 (0.32 ~ 0.66) | < 0.001 | 0.90 (0.71 ~ 1.13) | 0.360 |
| K-means clusters |  |  |  |  |  |  |
| Stable high | 1.00 (Reference) |  | 1.00 (Reference) |  | 1.00 (Reference) |  |
| Stable low | 0.81 (0.65 ~ 0.99) | 0.044 | 0.60 (0.42 ~ 0.85) | 0.004 | 0.92 (0.71 ~ 1.19) | 0.520 |
| Low to high | 1.00 (0.82 ~ 1.22) | 0.999 | 0.84 (0.62 ~ 1.15) | 0.272 | 1.17 (0.92 ~ 1.48) | 0.207 |
| High to low | 0.76 (0.63 ~ 0.92) | 0.004 | 0.55 (0.4 ~ 0.75) | < 0.001 | 0.90 (0.71 ~ 1.14) | 0.381 |

Data are presented as subdistribution hazard ratios (SHRs) and 95% confidence intervals (CIs). The Fine-Gray competing risk models were used to estimate the risk of CVD, stroke, and heart disease, treating all cause death as a competing event. Models were adjusted for age, sex, residence, education level, marital status, smoking status, drinking status, BMI, and baseline chronic conditions (hypertension, diabetes, dyslipidemia, CKD). Abbreviations: CVD, cardiovascular disease; CKD, chronic kidney disease; SHR, subdistribution hazard ratio; CI, confidence interval; Acc, accelerated; Non-Acc, non-accelerated.

**Table S5** Sensitivity analysis: Associations between KDM-BA dynamic metrics and the risk of incident CVD, stroke, and heart disease using Fine-Gray competing risk models

| Variables | CVD |  | Stroke |  | Heart Disease |  |
| --- | --- | --- | --- | --- | --- | --- |
|  | SHR (95%CI) | *P* | SHR (95%CI) | *P* | SHR (95%CI) | *P* |
| Δ KDM-BAacc |  |  |  |  |  |  |
| Continuous (per unit) | 1.03 (1.01 ~ 1.05) | 0.003 | 1.05 (1.02 ~ 1.09) | 0.003 | 1.03 (1.00 ~ 1.05) | 0.026 |
| Categorical |  |  |  |  |  |  |
| < 0 (decelerated) | 1.00 (Reference) |  | 1.00 (Reference) |  | 1.00 (Reference) |  |
| ≥ 0 (accelerated) | 1.27 (1.11 ~ 1.44) | < 0.001 | 1.42 (1.15 ~ 1.75) | 0.001 | 1.22 (1.05 ~ 1.42) | 0.010 |
| Aging rate |  |  |  |  |  |  |
| Continuous (per unit) | 1.13 (1.04 ~ 1.23) | 0.003 | 1.23 (1.07 ~ 1.41) | 0.003 | 1.12 (1.01 ~ 1.23) | 0.025 |
| Categorical |  |  |  |  |  |  |
| < 1 (Slow) | 1.00 (Reference) |  | 1.00 (Reference) |  | 1.00 (Reference) |  |
| ≥ 1 (Fast) | 1.21 (1.06 ~ 1.37) | 0.004 | 1.32 (1.07 ~ 1.64) | 0.010 | 1.16 (1.00 ~ 1.36) | 0.052 |
| Cumulative KDM-BAacc |  |  |  |  |  |  |
| Continuous (per unit) | 1.04 (1.01 ~ 1.07) | 0.004 | 1.10(1.05 ~ 1.15) | < 0.001 | 1.02 (0.99 ~ 1.05) | 0.243 |
| Tertiles |  |  |  |  |  |  |
| T1 | 1.00 (Reference) |  | 1.00 (Reference) |  | 1.00 (Reference) |  |
| T2 | 1.12 (0.94 ~ 1.33) | 0.207 | 1.07 (0.8 ~ 1.44) | 0.647 | 1.18 (0.97 ~ 1.45) | 0.104 |
| T3 | 1.23 (1.03 ~ 1.46) | 0.019 | 1.48 (1.11 ~ 1.98) | 0.008 | 1.16 (0.94 ~ 1.42) | 0.168 |
| Extreme phenotype |  |  |  |  |  |  |
| Anti-aging | 1.00 (Reference) |  | 1.00 (Reference) |  | 1.00 (Reference) |  |
| **Rapid** aging | 1.26 (0.97 ~ 1.65) | 0.089 | 1.85 (1.15 ~ 2.98) | 0.012 | 1.08 (0.79 ~ 1.48) | 0.630 |

Data are presented as subdistribution hazard ratios (SHRs) and 95% confidence intervals (CIs). The Fine-Gray competing risk models were used to estimate the risk of CVD, stroke, and heart disease, treating all cause death as a competing event. Models were adjusted for age, sex, residence, education level, marital status, smoking status, drinking status, BMI, and baseline chronic conditions (hypertension, diabetes, dyslipidemia, CKD). Abbreviations: CVD, cardiovascular disease; CKD, chronic kidney disease; SHR, subdistribution hazard ratio; CI, confidence interval; KDM-BAacc, Klemera-Doubal method biological age acceleration.

**Table S6** Sensitivity analysis: Associations of biological aging trajectories and clusters with the risk of incident CVD, stroke, and heart disease using multiple imputation for missing data

| Variables | CVD |  | Stroke |  | Heart disease |  |
| --- | --- | --- | --- | --- | --- | --- |
|  | HR (95%CI) | *P* | HR (95%CI) | *P* | HR (95%CI) | *P* |
| Longitudinal trajectories |  |  |  |  |  |  |
| Sustained acc | 1.00 (Reference) |  | 1.00 (Reference) |  | 1.00 (Reference) |  |
| Sustained non-acc | 0.77 (0.64 ~ 0.91) | 0.003 | 0.62 (0.47 ~ 0.82) | 0.001 | 0.86 (0.70 ~ 1.05) | 0.144 |
| Non-acc to acc | 0.98 (0.80 ~ 1.20) | 0.872 | 0.85 (0.62 ~ 1.18) | 0.336 | 1.16 (0.92 ~ 1.47) | 0.219 |
| Acc to non-acc | 0.72 (0.58 ~ 0.88) | 0.001 | 0.45 (0.32 ~ 0.65) | < 0.001 | 0.88 (0.70 ~ 1.12) | 0.310 |
| K-means clusters |  |  |  |  |  |  |
| Stable high | 1.00 (Reference) |  | 1.00 (Reference) |  | 1.00 (Reference) |  |
| Stable low | 0.77 (0.62 ~ 0.96) | 0.018 | 0.57 (0.40 ~ 0.81) | 0.002 | 0.89 (0.68 ~ 1.15) | 0.367 |
| Low to high | 0.98 (0.80 ~ 1.21) | 0.861 | 0.82 (0.60 ~ 1.13) | 0.237 | 1.15 (0.90 ~ 1.48) | 0.259 |
| High to low | 0.73 (0.60 ~ 0.89) | 0.002 | 0.53 (0.39 ~ 0.72) | < 0.001 | 0.87 (0.69 ~ 1.11) | 0.266 |

Data are presented as hazard ratios (HRs) and 95% confidence intervals (CIs). Multiple imputation by chained equations (MICE) was performed to account for missing covariate data. Models were adjusted for age, sex, residence, education level, marital status, smoking status, drinking status, BMI, and baseline chronic conditions (hypertension, diabetes, dyslipidemia, CKD). Abbreviations: CVD, cardiovascular disease; CKD, chronic kidney disease; HR, hazard ratio; CI, confidence interval; Acc, accelerated; Non-Acc, non-accelerated.

**Table S7** Sensitivity analysis: Associations between KDM-BA dynamic metrics and the risk of incident CVD, stroke, and heart disease using multiple imputation for missing data

| Variables | CVD |  | Stroke |  | Heart Disease |  |
| --- | --- | --- | --- | --- | --- | --- |
|  | HR (95%CI) | *P* | HR (95%CI) | *P* | HR (95%CI) | *P* |
| Δ KDM-BAacc |  |  |  |  |  |  |
| Continuous (per unit) | 1.03 (1.01 ~ 1.05) | 0.003 | 1.05 (1.02 ~ 1.09) | 0.002 | 1.03 (1.00 ~ 1.05) | 0.027 |
| Categorical |  |  |  |  |  |  |
| < 0 (decelerated) | 1.00 (Reference) |  | 1.00 (Reference) |  | 1.00 (Reference) |  |
| ≥ 0 (accelerated) | 1.28 (1.12 ~ 1.46) | < 0.001 | 1.43 (1.15 ~ 1.77) | 0.001 | 1.23 (1.05 ~ 1.44) | 0.009 |
| Aging rate |  |  |  |  |  |  |
| Continuous (per unit) | 1.13 (1.04 ~ 1.23) | 0.003 | 1.23 (1.08 ~ 1.40) | 0.002 | 1.12 (1.01 ~ 1.23) | 0.026 |
| Categorical |  |  |  |  |  |  |
| < 1 (Slow) | 1.00 (Reference) |  | 1.00 (Reference) |  | 1.00 (Reference) |  |
| ≥ 1 (Fast) | 1.23 (1.07 ~ 1.4) | 0.003 | 1.34 (1.08 ~ 1.67) | 0.008 | 1.18 (1.01 ~ 1.38) | 0.041 |
| Cumulative KDM-BAacc |  |  |  |  |  |  |
| Continuous (per unit) | 1.05 (1.02 ~ 1.07) | 0.001 | 1.1 (1.06 ~ 1.15) | < 0.001 | 1.02 (0.99 ~ 1.05) | 0.169 |
| Tertiles |  |  |  |  |  |  |
| T1 | 1.00 (Reference) |  | 1.00 (Reference) |  | 1.00 (Reference) |  |
| T2 | 1.13 (0.95 ~ 1.35) | 0.168 | 1.08 (0.81 ~ 1.45) | 0.597 | 1.20 (0.97 ~ 1.47) | 0.086 |
| T3 | 1.26 (1.06 ~ 1.50) | 0.010 | 1.52 (1.14 ~ 2.02) | 0.004 | 1.18 (0.95 ~ 1.45) | 0.126 |
| Extreme phenotype |  |  |  |  |  |  |
| Anti-aging | 1.00 (Reference) |  | 1.00 (Reference) |  | 1.00 (Reference) |  |
| **Rapid** aging | 1.34 (1.02 ~ 1.76) | 0.039 | 1.98 (1.23 ~ 3.19) | 0.005 | 1.13 (0.82 ~ 1.57) | 0.446 |

Data are presented as hazard ratios (HRs) and 95% confidence intervals (CIs). Multiple imputation by chained equations (MICE) was performed to account for missing covariate data. Models were adjusted for age, sex, residence, education level, marital status, smoking status, drinking status, BMI, and baseline chronic conditions (hypertension, diabetes, dyslipidemia, CKD). Abbreviations: CVD, cardiovascular disease; CKD, chronic kidney disease; HR, hazard ratio; CI, confidence interval; Acc, accelerated; Non-Acc, non-accelerated.

| Variables | CVD |  | Stroke |  | Heart disease |  |
| --- | --- | --- | --- | --- | --- | --- |
|  | HR (95%CI) | *P* | HR (95%CI) | *P* | HR (95%CI) | *P* |
| Longitudinal trajectories |  |  |  |  |  |  |
| Sustained acc | 1.00 (Reference) |  | 1.00 (Reference) |  | 1.00 (Reference) |  |
| Sustained non-acc | 0.52 (0.38 ~ 0.72) | < 0.001 | 0.28 (0.15 ~ 0.50) | < 0.001 | 0.69 (0.48 ~ 1.01) | 0.054 |
| Non-acc to acc | 0.77 (0.53 ~ 1.12) | 0.172 | 0.58 (0.31 ~ 1.09) | 0.090 | 0.96 (0.62 ~ 1.49) | 0.842 |
| Acc to non-acc | 0.64 (0.45 ~ 0.92) | 0.016 | 0.21 (0.09 ~ 0.50) | < 0.001 | 0.91 (0.61 ~ 1.36) | 0.645 |
| K-means clusters |  |  |  |  |  |  |
| Stable high | 1.00 (Reference) |  | 1.00 (Reference) |  | 1.00 (Reference) |  |
| Stable low | 0.52 (0.35 ~ 0.78) | 0.001 | 0.32 (0.16 ~ 0.65) | 0.001 | 0.63 (0.39 ~ 1.01) | 0.054 |
| Low to high | 0.79 (0.55 ~ 1.14) | 0.214 | 0.65 (0.36 ~ 1.18) | 0.156 | 0.88 (0.57 ~ 1.37) | 0.577 |
| High to low | 0.56 (0.40 ~ 0.80) | 0.001 | 0.29 (0.15 ~ 0.54) | < 0.001 | 0.72 (0.48 ~ 1.10) | 0.126 |

**Table S8** Sensitivity analysis: Associations of biological aging trajectories and clusters with the risk of incident CVD, stroke, and heart disease after excluding participants who developed outcomes during the first follow-up interval (Wave 4)

Data are presented as hazard ratios (HRs) and 95% confidence intervals (CIs). This sensitivity analysis excluded participants who developed CVD events between Wave 3 and Wave 4 to minimize potential reverse causality (lag analysis). Models were adjusted for age, sex, residence, education level, marital status, smoking status, drinking status, BMI, and baseline chronic conditions (hypertension, diabetes, dyslipidemia, CKD). Abbreviations: CVD, cardiovascular disease; CKD, chronic kidney disease; HR, hazard ratio; CI, confidence interval; Acc, accelerated; Non-Acc, non-accelerated.

**Table S9** Sensitivity analysis: Associations between KDM-BA dynamic metrics and the risk of incident CVD, stroke, and heart disease after excluding participants who developed outcomes during the first follow-up interval (Wave 4)

| Variables | CVD |  | Stroke |  | Heart Disease |  |
| --- | --- | --- | --- | --- | --- | --- |
|  | HR (95%CI) | *P* | HR (95%CI) | *P* | HR (95%CI) | *P* |
| Δ KDM-BAacc |  |  |  |  |  |  |
| Continuous (per unit) | 1.01 (0.97 ~ 1.05) | 0.652 | 1.03 (0.96 ~ 1.1) | 0.436 | 1.01 (0.96 ~ 1.05) | 0.775 |
| Categorical |  |  |  |  |  |  |
| < 0 (decelerated) | 1.00 (Reference) |  | 1.00 (Reference) |  | 1.00 (Reference) |  |
| ≥ 0 (accelerated) | 1.33 (1.04 ~ 1.69) | 0.022 | 1.48 (0.96 ~ 2.28) | 0.076 | 1.27 (0.96 ~ 1.68) | 0.100 |
| Aging rate |  |  |  |  |  |  |
| Continuous (per unit) | 1.04 (0.89 ~ 1.21) | 0.640 | 1.12 (0.85 ~ 1.47) | 0.423 | 1.03 (0.86 ~ 1.23) | 0.771 |
| Categorical |  |  |  |  |  |  |
| < 1 (Slow) | 1.00 (Reference) |  | 1.00 (Reference) |  | 1.00 (Reference) |  |
| ≥ 1 (Fast) | 1.16 (0.91 ~ 1.48) | 0.237 | 1.41 (0.91 ~ 2.18) | 0.126 | 1.06 (0.8 ~ 1.4) | 0.688 |
| Cumulative KDM-BAacc |  |  |  |  |  |  |
| Continuous (per unit) | 1.09 (1.04 ~ 1.15) | < 0.001 | 1.2 (1.11 ~ 1.31) | < 0.001 | 1.06 (1 ~ 1.12) | 0.063 |
| Tertiles |  |  |  |  |  |  |
| T1 | 1.00 (Reference) |  | 1.00 (Reference) |  | 1.00 (Reference) |  |
| T2 | 1.24 (0.89 ~ 1.74) | 0.200 | 1.14 (0.59 ~ 2.23) | 0.691 | 1.3 (0.89 ~ 1.89) | 0.173 |
| T3 | 1.64 (1.19 ~ 2.28) | 0.003 | 2.77 (1.53 ~ 5.02) | 0.001 | 1.38 (0.94 ~ 2.01) | 0.099 |
| Extreme phenotype |  |  |  |  |  |  |
| Anti-aging | 1.00 (Reference) |  | 1.00 (Reference) |  | 1.00 (Reference) |  |
| **Rapid** aging | 2.55 (1.46 ~ 4.47) | 0.001 | 6.43 (1.83 ~ 22.53) | 0.004 | 1.99 (1.05 ~ 3.77) | 0.034 |

Data are presented as hazard ratios (HRs) and 95% confidence intervals (CIs). This sensitivity analysis excluded participants who developed CVD events between Wave 3 and Wave 4 to minimize potential reverse causality (lag analysis). Models were adjusted for age, sex, residence, education level, marital status, smoking status, drinking status, BMI, and baseline chronic conditions (hypertension, diabetes, dyslipidemia, CKD). Abbreviations: CVD, cardiovascular disease; CKD, chronic kidney disease; CI, confidence interval; KDM-BAacc, Klemera-Doubal method biological age acceleration.
